# Quantum computing reduces systemic risk in financial networks

**DOI:** 10.1038/s41598-023-30710-z

**Published:** 2023-03-09

**Authors:** Amine Mohamed Aboussalah, Cheng Chi, Chi-Guhn Lee

**Affiliations:** 1 Department of Financial and Risk Engineering, New York University, New York, USA; 2 Department of Mechanical and Industrial Engineering, University of Toronto, Toronto, Canada

**Keywords:** Mathematics and computing, Applied mathematics, Computational science, Computer science, Information technology, Scientific data, Quantum information, Quantum simulation, Qubits

## Abstract

In highly connected financial networks, the failure of a single institution can cascade into additional bank failures. This systemic risk can be mitigated by adjusting the loans, holding shares, and other liabilities connecting institutions in a way that prevents cascading of failures. We are approaching the systemic risk problem by attempting to optimize the connections between the institutions. In order to provide a more realistic simulation environment, we have incorporated nonlinear/discontinuous losses in the value of the banks. To address scalability challenges, we have developed a two-stage algorithm where the networks are partitioned into modules of highly interconnected banks and then the modules are individually optimized. We developed a new algorithms for classical and quantum partitioning for directed and weighed graphs (first stage) and a new methodology for solving Mixed Integer Linear Programming problems with constraints for the systemic risk context (second stage). We compare classical and quantum algorithms for the partitioning problem. Experimental results demonstrate that our two-stage optimization with quantum partitioning is more resilient to financial shocks, delays the cascade failure phase transition, and reduces the total number of failures at convergence under systemic risks with reduced time complexity.

## Introduction

Modern financial markets are experiencing an increase of interdependencies among organizations such as banks, companies, or even countries. These interdependencies are in form of loans, holding shares, and other liabilities. Institutions connected by their interdependencies form the financial network. Such connections can amplify financial shocks on the underlying assets, spread financial contagion, and lead to more failures of institutions in the network^1^. This is known as cascade failure and it can lead to catastrophic consequences to economies and people’s lives. For instance, the 2008 Global Financial Crisis was caused by the occurrence of cascade failures among banks in the North American market due to mortgage default shocks.

Cascade failures of financial systems have been studied for many years. An equation for calculating the market value of banks based on their linear interdependencies and asset holdings was developed by Brioschi et al.^2^ Elliott et al.^3^ built a financial network model using the same linear equation and introduced discontinuous value loss for institutions when their values drop below a critical level. They also studied and simulated cascade failures through linear interdependencies in their network model. When the number of institutions is large, keeping track of cascade failure through the network becomes an intractable problem. Orus et al.^4^ proposed a quantum version of the bank values calculation using the equation proposed by Elliott et al.^3^ These models of interbank networks and cascade failures share some common assumptions: values of institutions ultimately depend on the values of primitive assets that are independent from each other, and the interdependencies among institutions are linear. Under these common assumptions, cascade failures have been studied in financial networks with various structures of interdependencies such as core-periphery structure and sector-segregation structure^3^.

Diem et al.^5^ proposed a mixed integer programming algorithm that can directly optimize the liabilities between banks so that the total losses are minimized under financial shock. The interbank network model by Diem et al.^5^ does not include discontinuous value loss which brings nonlinearity to the network and breeds cascade failures. Their results do not show how the network optimization could improve the network resilience to the cascade failures.

Capponi and Chen^6^ described the mitigation of systemic risk by providing liquidity assistance in a multi-period clearing framework. They studied the sensitivity of systemic risk in relation to interbank liabilities and their correlation structure. Nier et al.^7^ investigated how the structure of the financial system affects systemic risk. They varied four important parameters: capitalization, connectivity, interbank exposures, and concentration. In addition, they investigated liquidity effects and asymmetry in the banking structure. Roncoroni et al.^8^ studied the interaction between the financial network structure and the market conditions in the context of systemic risk. They draw a distinction between direct and indirect interconnectedness and study the impact of diversification in portfolio allocations. Hué et al.^9^ proposed a new measure of systemic risk that combines the pair-wise Granger causality approach and the leave-one-out concept. Their experimental results show that the size and the investment strategies of banks are significant drivers of systemic risk. Lux^10^ proposed a bipartite credit network model where non-bank corporate entities are connected to a network of banks. While basic characteristics do not seem to predict the contagion, joint exposures to corporate entities were found to be more important than interbank credit. Ramadiah et al.^11^ tested systemic risk in bipartite networks with different network reconstruction strategies and found that the original network displayed more systemic risk than any of the reconstructed networks. So et al.^12^ proposed a dynamic topic network (DTN) approach that combines network analysis and topic modeling to evaluate systemic risk and provide indication of abnormal movements in financial markets.

Birge^13^ presents an alternative viewpoint that organizations are rational agents that seek to maximize their expected utility in the Markowitz portfolio sense, which means that the cross-holdings are calculated from a local agent’s perspective and therefore do not take into account mitigation of systemic risk. He studied how organizations respond to systemic risk by evaluating various financial parameters using real world data. In our work, we directly optimized the cross-holdings between organizations from a global regulator’s perspective in order to mitigate systemic risk of the global network in the face of exogenous shocks. The reinforcement learning agents in^14^ and^15^ would be good candidates for extending^13^ work to use multi-agent learning to better understand and mitigate systemic risk.

The nonlinearity introduced in the interbank network model by Elliott et al.^3^ is a crucial feature since it models the loss of public confidence or other abrupt changes to the value of the bank, which leads to cascade behaviors of the interbank network similar to the cascade failures in the 2008 financial crisis. As far as we know, no study has been done that directly targets mitigating this highly nonlinear cascade failure, and we aim to fill this gap. The network optimization model proposed by Diem et al.^5^ provides solutions with drastic changes in the connectivity, which may not be feasible in practice. In addition, it is also not possible to use the Diem et al.^5^ model to optimize large realistic interbank networks due to scalabilty issues. We have developed an optimization model to strengthen the interbank network’s resilience to cascade failure that can easily scale up and optimize interbank networks with a large number of banks. To test the performance of our optimization algorithm, we developed a computational systemic risk environment for cascade simulations based on the Elliott et al.^3^ model with the nonlinear value loss. There are one-stage and two-stage versions of our optimization model where the extra stage is network partitioning. We experimented with both classical and quantum partitioning. The two-stage optimization model with quantum partitioning performs the best in delaying cascade failures.

Herman et al.^16^ presents a thorough review of quantum computing for solving computationally challenging financial problems. Several optimization problems in finance, as well as machine learning algorithms that could potentially benefit from quantum computing are covered. Many financial applications such as portfolio management, derivative pricing, risk modeling, and collaterialized debt obligation (CDO) are described. More specifically, the CDO section mentions how default risks are connected to systemic risk and explains how they can lead to the collapse of the entire economy like what happened in the 2008 financial crisis. The CDO models treats systemic risk as an exogoneous random latent variable and studies its correlation with the default risks. Our paper computes systemic risk using an agent based model and aims to find optimal actions to take from a regulator perspective to control systemic risk.

Grant et al.^17^ benchmarks the empirical results of the optimal solutions for a variety of Markowitz portfolio optimization instances against the computational ground truth solutions using the D-Wave 2000Q quantum annealer. There are several controls used in programming quantum annealers that may impact the probability of success. They studied the effects of multiple controls and reported those that yield a higher probability of sucess. This work deals with the portfolio theory problem from a quantum computing perspective with a local point of view and a local understanding of risk captured by the Markowitz portfolio theory. The collective behavior of many individual portfolio managers gives rise to global risks that cannot be evaluated or mitigated by these portfolio managers. Our paper considers a global viewpoint to address these systemic risks.

In section "Computational systemic risk environment" we introduce our computational systemic risk environment. We discuss our one-stage optimization model in section "Interbank network optimization model", and our two-stage optimization model and its quantum version in section "Optimization model with network partitioning". In section "Performance and scalability" we present our experimental results of cascade mitigation using synthetic data and in section "Experimental results with real-world data" we present experimental results with real-world data.

## Computational systemic risk environment

### Interbank network value calculation

The computational interbank systemic risk environment that we built is based upon the bank value equation formulated by Elliott et al.^3^:

1 $$\begin{aligned} \vec {v} = \hat{ C } ( I - C )^{-1}\Big ( D \vec {p}-\vec {b}(\vec {v},\vec {p})\Big ) \end{aligned}$$

where $\vec {v} \in {\mathbb {R}}^{N}$ is the value vector of banks specifying the value of each bank where *N* is the total number of banks; $C \in {\mathbb {R}}^{N \times N}$ is the cross-holding matrix where entry $C_{ij}$ specifies the percentage of bank *j* held by *i*, which is also the strength of the linear dependency between bank *i* and bank *j*. $\hat{ C } \in {\mathbb {R}}^{N \times N}$ is the self holding matrix, which specifies the percentage of the bank value that is actually held by itself, where $\hat{C_{ii}}$ equals $1-\sum _{j}C_{ji}$. The self holding matrix is used to discount the value of banks so that the calculated $\vec {v}$ becomes the non-inflated “market” value instead of the inflated “equity” value^18^. $D \in {\mathbb {R}}^{N \times N_a}$ is the assets-holding matrix, where entry $D_{ij}$ specifies the percentage of asset *j* held by bank *i* and $N_a$ is the total number of assets. $\vec {p} \in {\mathbb {R}}^{N_a}$ is the primitive price vector of assets and $\vec {b} \in {\mathbb {R}}^{N}$ is the nonlinear penalty vector representing the loss of public faith in bank *i* if it crashes. This nonlinearity opens up the opportunity for chaotic behaviors of the network under financial shocks, and it contributes to the overall systemic risk in the interbank network. Notice that bank *i* crashes only when its value drops below a critical threshold value, which we set to be a fixed percentage of the bank’s initial value.

### Interbank network generation

For testing, we generate an interbank network in the environment with randomized synthetic data. Then, we optimize the interbank network using our algorithm. For both the original network and the optimized network, we apply a shock and simulate the propagation of the shock through the network. We can then compare the results between the original network and the optimized network over multiple trials.

We randomly generate each entry $C_{ij}$ to be between 0 and 1. However, there are two extra considerations. First, the summation of each column of the $C$ matrix must be smaller than 1, as bank *i*’s self-holding $\hat{ C _{ii}}$ (equals $1-\sum _{j} C _{ji}$) must be positive. Second, random generation will lead to a fully connected network, and it is rare that all the banks in real life are directly connected through cross-holdings. Therefore, we set a threshold value (for instance, 0.005) for $C _{ij}$ so that if the generated $C _{ij}$ is smaller than this threshold, we set $C_{ij}$ to be 0 for this particular *i* and *j*. We generate each entry $D _{ij}$ randomly between 0 and 1, while keeping the $\sum _{i} D _{ij}$ to be 1 as each asset is completely owned by banks. We use a similar threshold technique as in the cross-holdings generation case to keep the assets-holding relatively concentrated. We randomly generate $\vec {p}$ with values between 0 and 1.

**Figure 1:**
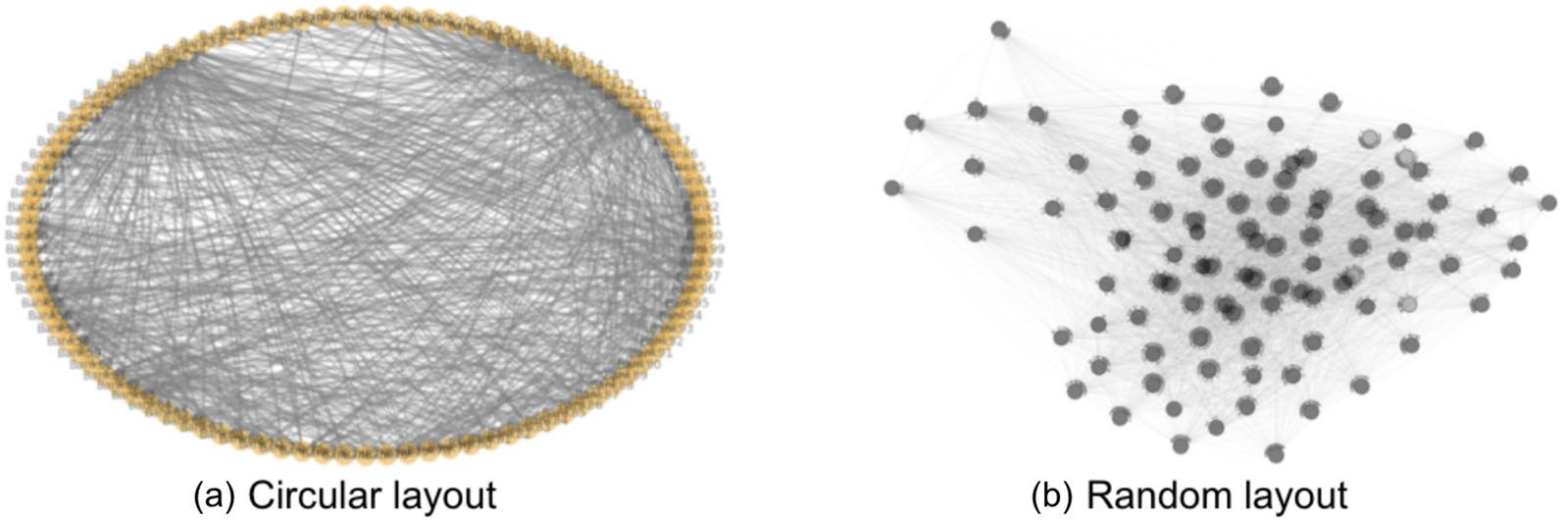
Example interbank network visualizations. (**a**) shows bank nodes in a circular layout. (**b**) shows bank nodes in a random layout. We use these two layouts interchangeably for better illustration.

Figure 1 is the visualization of an interbank network model that contains 100 banks with randomly generated dependencies. Two different visualization layouts show the same network: 1.(a) is the circular layout and 1.(b) is the random layout. In the circular layout on the left, the nodes, which are positioned circularly, represent banks, and these banks are connected by cross-holdings, shown by the arrows within the circle formed by nodes. In the random layout on the right, the nodes represent the banks, and the arrows between nodes specify the cross-holdings among banks. In both layouts, the thickness of the arrows connecting nodes represents the strength and direction of cross-holding between those two banks. The cross-holding between bank *i* and bank *j* is specified by the value of $C_{ij}$.

### Financial shock and cascade failures simulation

Based upon the network model shown in Fig. 1, financial shocks are simulated to cause cascade failures within the interbank network, and the total number of crashes in the network given a certain amount of shock is our measure of the level of systemic risk. A higher number of crashes indicates a higher level of systemic risk. This simulation is modeled by an iterative algorithm where the newly failed banks in the current layer will be the input to the next iteration layer. The core equation is again Eq. (1) where the values of banks within the network structure shown in Fig. 1 are calculated. Bank$_{i}$ is said to fail if its value $v_{i}$ ($i_{th}$ element of $\vec {v}$) drops below a critical value $v^c_{i}$ that is smaller than $v_{i}$. Once bank *i* fails, it suffers an additional value loss $b_{i}$($i_{th}$ element of $\vec {b}$). The perturbations that start cascade failures are assets perturbations. We perturb the assets held by banks by decreasing the value of those assets, and the perturbation is characterized by parameters $\alpha$ and $\beta$. Parameter $\alpha \in [0,1]$ is the perturbation amplitude, and the higher the $\alpha$, the more we decrease the value of the assets. With n being total number of banks, parameter $\beta \in \{0,\ldots ,n\}$ specifies the number of assets whose value would be decreased in this perturbation.
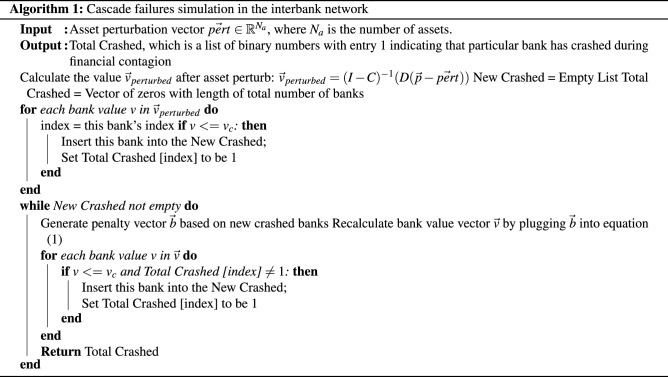


The full cascade simulation algorithm is shown as pseudo code in Algorithm 1. The cost incurred by a defaulted or failed bank is 21.7% of the market value of an organization’s assets on average^3,19^. Therefore, the penalty vector $\vec {b}$ for crashed banks is generated such that the vector elements corresponding to the crashed banks are assigned values of 21.7% of the initial values of those banks, and all other non-crashed elements are zero. The procedure in Algorithm 1 before the while loop is for calculating the initial crashed banks due to the asset perturbation, or in other words, the sources of the cascade. Within the while loop, the cascade process of the interbank network is simulated.

**Figure 2:**
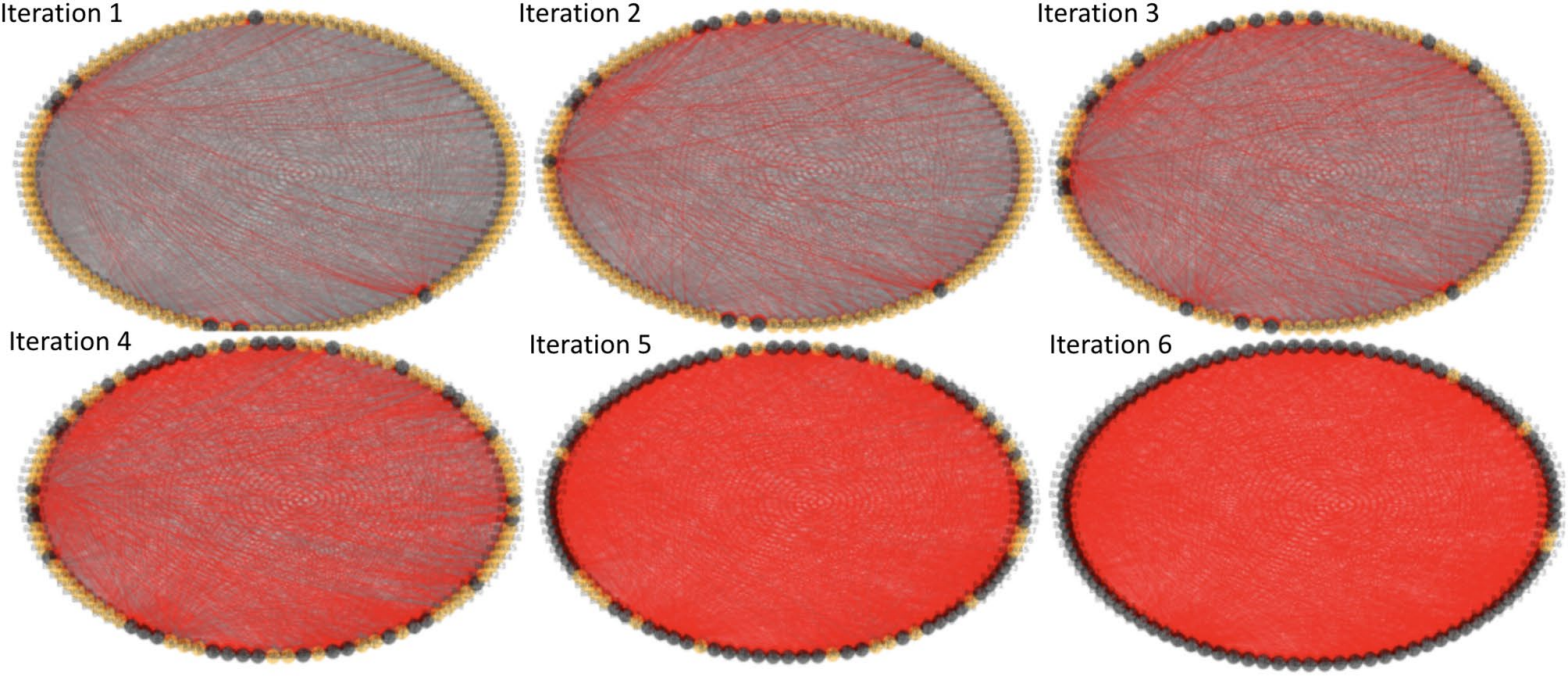
Cascade simulation visualization for an interbank network with 100 banks, which converged after 6 iterations. Banks are shown as orange nodes, failed banks are shown as black nodes, and failed banks will incur further loss to other banks through cross-holdings shown as red edges. The red edges can be interpreted of as the pathways of financial contagion or loss propagation. The cascade process is triggered by asset perturbation before iteration 1.

Figure 2 shows an example run of this cascade failure simulation on an interbank network with 100 banks and 50 assets with a circular layout. The initial asset perturbation for this example has $\alpha =0.7$ and $\beta =24$. As a result of this perturbation, 97 out of 100 banks crashed after 6 iterations, and the interbank network has crashed.

**Figure 3:**
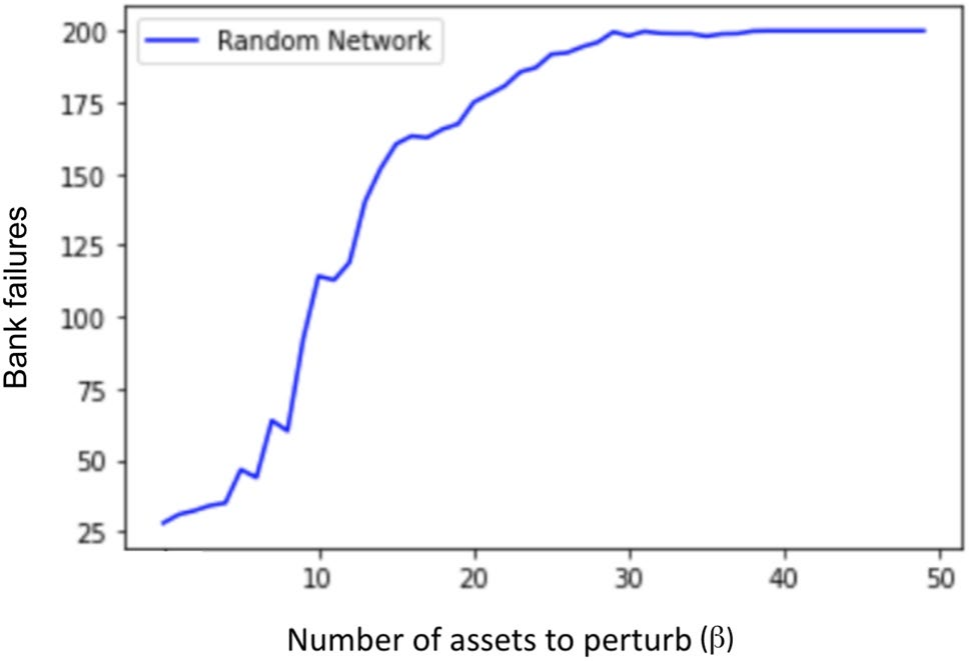
Average cascade simulation results showing bank failures relative to an increasing number of perturbed assets ($\beta$). For each $\beta$, 5 random interbank networks are generated. We generate an asset perturbation to start the cascade, record the total number of bank failures after the cascade simulation terminates, take the average of number of failures of the 5 networks, and plot that average number on the y-axis for the given $\beta$.

To get more general cascade simulation results, we vary the number of perturbed assets ($\beta$) input to Algorithm 1, and for each beta, we generate 5 different random networks with 200 banks and take the average of the number of total failures after convergence. The result is shown in Fig. 3.

As we can see in Fig. 3, when $\beta$ (number of perturbed assets) is larger than 5, there is a drastic increase in the number of crashed banks (a phase transition) and soon all the banks fail in the network. In real life, such a cascade with almost all banks failing could have catastrophic consequences. Therefore, our goal is to optimize the network structure so that the whole interbank network is more resilient to financial shocks and less failures would be incurred during the cascade process. In other words, we hope to postpone the cascade shown in Fig. 3 and right shift the phase transition of failures occurring here when $\beta$ is around 5 to larger beta values. To achieve this, we first develop a one-stage optimization algorithm which we will describe in section "Interbank network optimization model", and then based on this algorithm, we develop an algorithm that has better efficiency called two-stage optimization that we will describe in section "Optimization model with network partitioning".

## Interbank network optimization model

### Mitigating systemic risk with network optimization

Our goal in this research is to understand and mitigate systemic risk in the interbank network. We want to modify the interbank network structure to make it more resilient to financial shocks. To achieve this, an optimization algorithm that minimizes the losses that can occur due to the crashes of banks can be used. Our computational systemic risk environment allows us to compute a quantitative measure of the severity of cascade failures through multiple trials using Algorithm 1. Minimizing this quantitative measure via network optimization should mitigate cascade failures of the interbank network. Implementation of the optimized network will require banks to adjust the amount of loan, holding, and other liability connections.

### Mixed Integer Linear Programming formulation

Inspired by the work done by Diem et al.^5^, we formulate the nonlinear interbank network structure optimization problem as a Mixed Integer Linear Programming (MILP) problem. As described in section "Mitigating systemic risk with network optimization", we are minimizing the total value loss of all banks during the cascade process. The loss of bank value is directly due to the penalty vector $\vec {b}$ in Eq. (1) and we seek to minimize this loss. The nonlinear optimization model is shown in Eqs. (2)–(4):

2 $$\begin{aligned} \min _{C_{ij}} \quad \sum _{j}\sum _{i}\min \left( \dfrac{C_{ij}*\gamma *v_{j}}{v_i},1\right) \end{aligned}$$

3 $$\begin{aligned} \text {s.t.} \quad \sum _{j}C_{ij}*v_{j} = e_i \quad \forall i \end{aligned}$$

4 $$\begin{aligned} \quad \sum _{j}C_{ji} = \sum _{j}C^{init}_{ji} \quad \forall i \end{aligned}$$

Equation (2) is the objective function that we are minimizing. $\gamma$ is a parameter set to be 0.217, and it corresponds to the fact that bank *i* loses 21.7% of its value when it crashes as described in section "Financial shock and cascade failures simulation". As $C_{ij}$ specifies the percentage of bank *j* that is owned by bank *i*, the quantity $\gamma *C_{ij}*v_{j}$ indicates the loss of bank *i* due to the failure of bank *j*. The inner sum over *i* is the total loss of all banks in the network due to the failure of bank *j*, therefore, the outer sum over *j* is the total loss of all banks in the network due to failures of all the other banks. Our goal is to minimize the total possible loss in the network during cascade failures. The value of bank *i*, $v_{i}$ in the denominator and the minimum operator ensures that the bank *i* can never have a loss larger than its total value. The objective function is piece-wise linear and concave because of the minimum operator, and the sum of concave functions is concave, thus the optimality remains under the minimum operator.

The quantity $e_{i}$ is the exposure of bank *i*, which is bank *i*’s total holdings of other banks calculated as $\sum _{j}C^{init}_{ij}*v_{j}$, where $C^{init}$ is the cross-holding matrix of the interbank network before the optimization. The constraint in (3) ensures that the total exposure of each bank remains constant. The constraint in (4) ensures that the proportion of the bank *i*’s value held by itself remains unchanged. Even though the systemic risk can be minimized if banks become disconnected and stop holding other’s shares, as there would be no link for the cascade to spread, it is unrealistic to ask banks to increase their self-holding level to 100%.

Therefore, our optimization algorithm aims to mitigate cascade failures only by rearranging banks’ cross-holdings without changing their total liabilities and their overall financial structures.

To remove the minimum operator in the objective function in (2), we expanded it into a MILP optimization model. The auxiliary decision variables $temp _{ij}$, binary variables $y1 _{ij}$, $y2 _{ij}$ and two constraints are added to replace the minimum operator. The MILP formulation is shown in Eqs. (5)–(10):

5 $$\begin{aligned} \min _{ temp , y1 , y2 , C } \quad \sum _{j}\sum _{i} temp _{ij} \end{aligned}$$

6 $$\begin{aligned} \text {s.t.} \quad {\text {temp}}_{ij} >=\frac{\gamma * C _{ij}*v_{j}}{v_i} - M* y1 _{ij} \quad \forall i,j \end{aligned}$$

7 $$\begin{aligned} \quad {\text {temp}} _{ij} >= 1 - M* y2 _{i,j} \quad \forall i,j \end{aligned}$$

8 $$\begin{aligned} \quad y1 _{ij} + y2 _{i,j} = 1 \quad \forall i,j \end{aligned}$$

9 $$\begin{aligned} \quad \sum _{j} C _{ij}*\gamma *v_{j} = e_i \quad \forall i \end{aligned}$$

10 $$\begin{aligned} \quad \sum _{j} C _{ji} = \sum _{j} C ^{init}_{ji} \quad \forall i \end{aligned}$$

Decision variables *y*1, *y*2, and ${\text {temp}}$ are added as auxiliary variables to remove the *min* operator in the original objective function given by Eq. (2), with $y1_{i,j}$, $y2_{i,j}\in \{0,1\}$ and ${\text {temp}}_{i,j} \in {\mathbb {R}}$. These sets of three auxiliary variables have the same size as decision variables $C$, which is $N(N-1)$ where *N* is the total number of banks in the network. With *M* being a big number, constraints (6)–(8) ensure the equivalence of ${\text {temp}}_{i,j}$ to $\min {\left( \frac{\gamma * C _{ij}*v_{j}}{v_i},1\right) }$ in Eq. (2) in the following way: For each {i,j}, $y1 _{i,j} + y2 _{i,j} = 1$ requires that exactly one binary equals 1, so that exactly one constraint out of (6) and (7) is no longer active. Therefore, $temp _{i,j}$ is either greater than or equal to $\min {\left( \frac{\gamma * C _{ij}*v_{j}}{v_i},1\right) }$ or 1, and since the algorithm solver is minimizing the objective function, it will only try to fix it to the minimum of these two values to ensure optimality.

**Figure 4:**
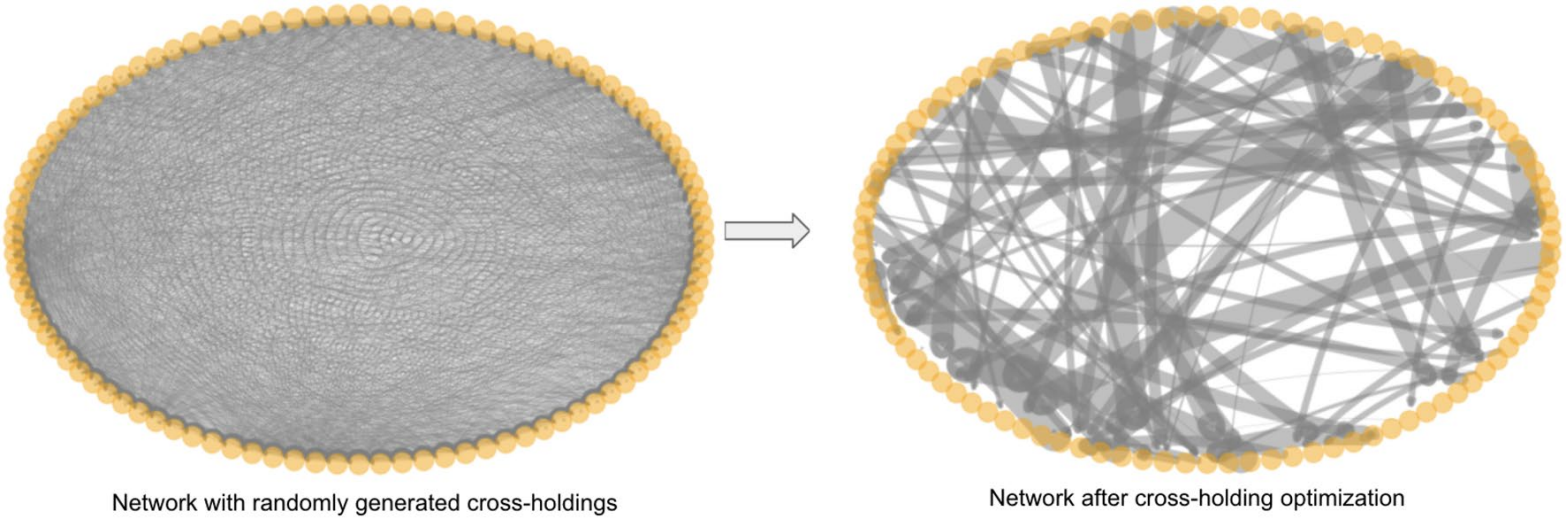
Example interbank network using one-stage optimization. The left plot is the circular layout visualization of a randomly generated interbank network with 100 banks. The right plot is the network after cross-holding optimization.

This MILP algorithm described above would return the optimal $C$, which indicates the optimal cross-holdings between banks. One example of the changes of the cross-holdings from a random network to an optimal network with 100 banks is shown in Fig. 4. From Fig. 4 we can see that there is a contraction of the cross-holdings after the optimization. One potential issue about such optimization is that it requires relatively large changes of bank connections from the current cross-holding structure, which could be unrealistic in real life. This issue can be reduced using our two-stage optimization method which we discuss in section "Optimization model with network partitioning". Nevertheless, this current optimization algorithm is able to select and enhance certain cross-holdings in a non-trivial way while decreasing other cross-holdings to achieve the most cascade-resilient structure.

### The curse of dimensionality

The decision variables in the classical MILP algorithm are sets of $C_{ij}$, ${\text {temp}}_{ij}$, $y1_{ij}$, and $y2_{ij}$ each with size $N(N-1)$ with *N* being number of banks. Therefore, the total number of decision variables grows quadratically with the size of the network. In general, the complexity of optimization problems grows drastically with the number of decision variables, which again grows quadratically in the size of the network. Accordingly, this problem could become intractable using the one-stage optimization algorithm when the size of the interbank network is large, which is often the case for real life banking networks. For instance, there are around 5,000 commercial banks currently in North America according to the FDIC quarterly report by the Federal Deposit Insurance Corporation^20^.

Facing such complexity issues in a large interbank network, we apply the notion of network modularity or communities to partition the whole interbank network into different modules where banks within each module generally have high connectivity. Then we apply the same optimization to each individual module. A large optimization problem is decomposed into multiple small optimization problems. In this way, the size of the optimization problem can be reduced and the curse of dimensionality can be mitigated.

## Optimization model with network partitioning

To overcome the curse of dimensionality described in section in "The curse of dimensionality" and unrealistic cross-holding changes in section "Mixed Integer Linear Programming formulation", we incorporate network community partitioning into our interbank network optimization algorithms. In doing so, we convert our one-stage optimization for the whole network into optimizations of multiple modules (communities). This multiple module optimization procedure consists of two stages: first find modules of the interbank network, then optimize cross-holdings in each of module. Therefore, we define this multiple module optimization as a two-stage optimization. We state our motivation and intuition behind two-stage optimization in section “Motivation for using network modularity”. Then we introduce modularity optimization algorithms for network community partitioning in section "Defining network modularity", define modularity for interbank network in section “Defining interbank network modularity”, define Ising formulation for interbank network modularity optimization in section “Ising formulation for network modularity optimization”, and discuss in detail our two-stage optimization in section “Two-stage optimization algorithm”.

### Motivation for using network modularity

The MILP optimization is aimed at finding the optimal connections of banks within the network that minimize the incurred loss due to bank failures. As a reminder, the decision variables are $C _{ij}$, which are the percentages of bank *j* held by *i* or liability connections. Applying community partitioning to the interbank network before optimization is a way to decrease the size of the MILP problem, as instead of solving a large MILP containing all the banks, we solve several smaller MILPs each corresponding to a different module or community within the whole interbank network. In doing so, we are optimizing only a subset of decision variables $( C _{ij})$ and leaving the the cross-holdings between banks in different communities (modules) unchanged. Therefore, one natural question arises: would such a reduction of the optimization problem lead to worse performance in terms of mitigating cascade failure?

Comparison of the experimental results for the original one-stage optimization and the two-stage optimization indicates that their performance in delaying the cascade failures is similar but the computational time requirement is significantly reduced as shown in section "Performance and scalability". Here we only discuss our intuition of why they have similar performance and why only optimizing cross-holdings of banks in the same community (module) is intuitive.

The cascade failure, which is modeled by Algorithm 1, originates from the sudden drop of assets’ values due to assets perturbation. With the sudden drop of values of the assets, banks that hold those assets suffer a drop in their values, and some banks’ values drop below their critical thresholds and incur discontinuous value losses for banks. Those discontinuous value losses, leading to more losses and failures, would be the start of the cascade failure. Within iterations of the cascade failure of Algorithm 1, the cascade failure reinforces itself in the sense that the crashed banks from the previous iteration would become the source of more bank failures in the next iteration and so on. Intuitively, the best way to mitigate such a self-reinforcing chain of failures would be confining failures at the initial few iterations. At the beginning, only a few banks have crashed, and the incurred value losses are only spreading in a local scale within the banks that are highly connected with the crashed banks. These highly connected groups of banks, or banks that are highly dependent in terms of liabilities, are exactly what constitute modules or communities of the interbank network. Therefore, to confine the cascade at the beginning is to confine the cascade within the local modules or communities, which is our overall strategy to mitigate cascade failures of the interbank network.

**Figure 5:**
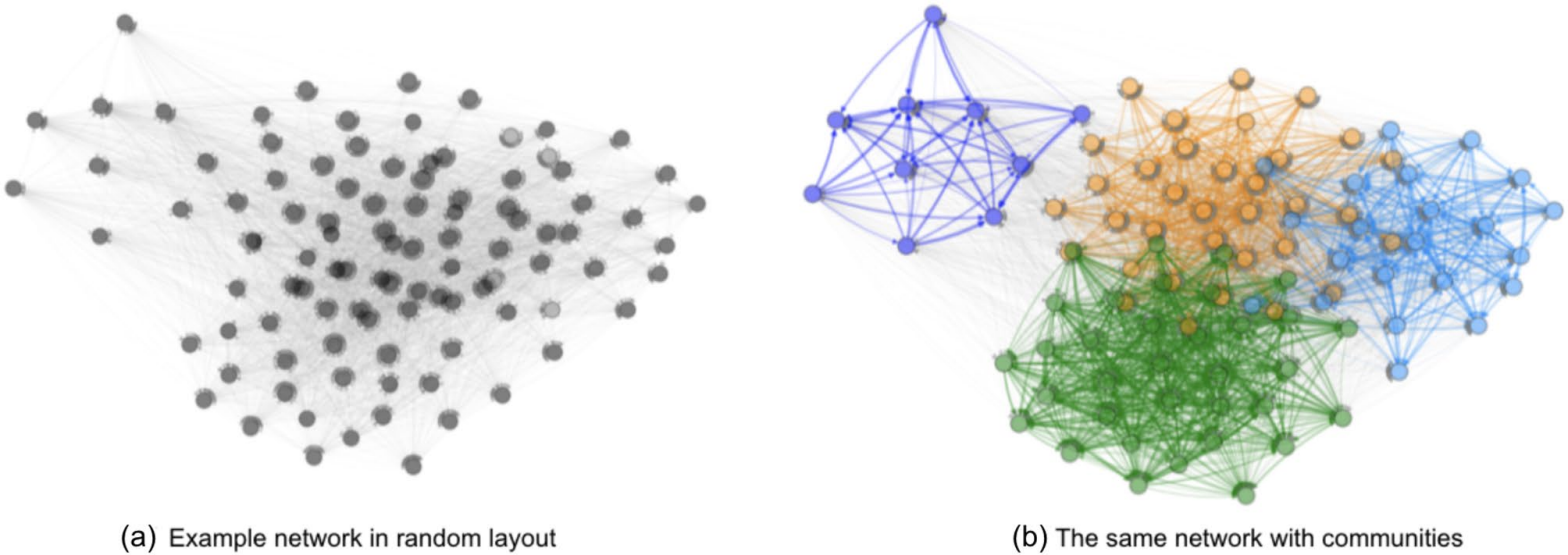
Two figures show the same randomly generated interbank network, where (**b**) shows the communities of the network in (**a**) computed using interbank modularity.

With the reasons discussed above, applying community partitioning to the interbank network and then optimizing the liability structures of banks within each module becomes intuitive and highly plausible. Figure 5 shows an example of communities in an interbank network. The interbank network is shown in random layout for a better visualization of the community structure.

**Figure 6:**
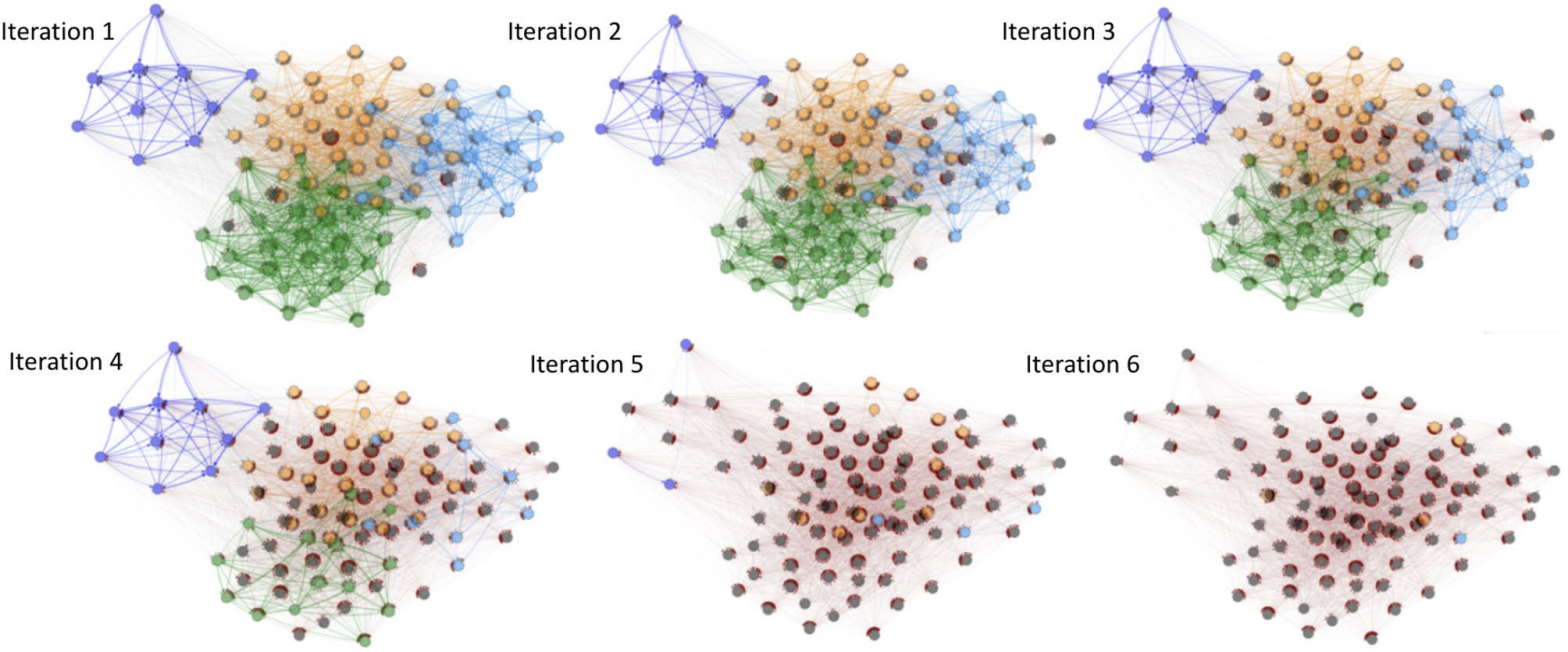
In this cascade simulation visualization, banks are shown in nodes with different colors corresponding to different communities, and failed banks are shown in black. Red edges can be interpreted as the pathways of loss propagation similar to Fig. 2. Notice in this visualization of cascade that financial contagion occurs within each community first, which motivates our two-stage optimization.

The interbank network example shown in Fig. 5 is the same network shown in Fig. 1. Figure 5b on the right shows the community structure of this interbank network example, where different communities or modules are shown in different colors. Figure 6 shows the cascade simulation on the example interbank network, which is the same cascade simulation process in Fig. 2. This cascade simulation visualization validates the insights we discussed in this section, as we can see that cascade failures indeed spread through communities or modules first. Therefore, only optimizing the cross-holdings of each module should suffice in confining cascade failures and stop the waves of bank failures, which is also supported by our experimental results shown in section "Performance and scalability" later.

Now that we have discussed the critical role that modules can play in controlling cascade failures, let us turn to a few algorithms that can be used to detect the communities or modules in a network in section "Defining network modularity".

### Defining network modularity

#### Definition 1

(Graph definition) A *graph* ${\mathbb {G}} = ({\mathbb {V}}, {\mathbb {E}})$, consists of a finite set of vertices (nodes) ${\mathbb {V}} = \{v_{1},\ldots , v_{k}\}$ and a finite set of edges ${\mathbb {E}} \subseteq {\mathbb {V}} \times {\mathbb {V}}$ connecting the verticies. Vertices $v_{m}$ and $v_{n}$ are connected by an edge if and only if $(v_{m}, v_{n}) \in {\mathbb {E}}({\mathbb {G}})$.

#### Definition 2

(Graph terminology) Graph ${\mathbb {G}} = ({\mathbb {V}}, {\mathbb {E}})$ is an undirected graph if elements in ${\mathbb {E}}$ indicate a two-way relationship, in that each edge can be traversed in both directions. Graph ${\mathbb {G}} = ({\mathbb {V}}, {\mathbb {E}})$ is a directed graph if elements in ${\mathbb {E}}$ indicate a one-way relationship, in that each edge can only be traversed in a single direction. Graph ${\mathbb {G}} = ({\mathbb {V}}, {\mathbb {E}})$ is a weighted graph if each element in ${\mathbb {E}}$ is given a numerical weight.

#### Definition 3

(Community definition) A community ${\mathbb {C}}$ with respect to graph ${\mathbb {G}}$ is defined as a subset of nodes that are densely connected to other nodes in ${\mathbb {C}}$ and loosely connected to the nodes in the other communities in the same graph ${\mathbb {G}}$.

For an interbank network, connectivity is not uniformly distributed throughout the network. Nodes within the same community or module have higher connectivity compared to nodes belonging to a different community. More specifically, if two nodes belong to same module or community, they are more likely to be connected.

Community structure is a common characteristic of networks and it has been studied in depth in graph theory^21–24^. Community partitioning is a task of extracting communities defined in Definition 3 from large networks. One approach for community partitioning is to optimize the modularity, which indicates the quality of the communities detected. The Louvain algorithm, developed by Blondel et al.^25^, is a particular greedy optimization method for modularity optimization that iteratively updates communities to produce the largest increase in modularity. Being a heuristic-based optimization algorithm, it does not provide an optimality guarantee for the communities it finds, and it generally requires the user to specify the convergence criterion. In our implementation, we terminate the algorithm if the modularity gain between two iterations is less than 1e-5. When applying the Louvain algorithm for directed graphs (defined in Definition 2), a common approach is to simply forget the edge direction when detecting communities in a large network and to run Louvain’s algorithm on the simplified undirected network^25^, which is what we did in our classical network partitioning in our experiments.

For a non-weighted, non-directed network ${\mathbb {G}}$, the modularity (called ${\mathscr {Q}}$) is defined in Eq. (11):

11 $$\begin{aligned} {\mathscr {Q}} = \quad \frac{1}{2m}\sum _{ij} \left[ A _{ij}-\frac{d_{i}d_{j}}{2m}\right] \delta {(c_{i},c_{j})} \end{aligned}$$

where ${ A _{ij}}$ is the matrix element for the adjacency matrix $A$, and ${ A _{ij}} = 1$ represents that there is an edge between node *i* and node *j*, and 0 otherwise. *m* is the total number of edges within the non-weighted non-directed ${\mathbb {G}}$. $d_i$ is the number of nodes that are connected to node *i*. $c_i$ is the community of node *i*, and the delta function $\delta {(c_{i},c_{j})}$ is defined to be 1 if node *i* and *j* belong to the same community, and 0 otherwise.

The intuition behind this definition is the following: we want to maximize ${\mathscr {Q}}$ by assigning node *i* and *j* to be in the same community ($\delta {(c_{i},c_{j})} = 1$) when the term $A _{ij}-\frac{d_{i}d_{j}}{2m}$ is relatively large. The term would be large if there is an edge between node *i* and *j* ($A _{ij}$=1) while node *i* and *j* do not have a large degree of connections to other nodes in the graph ($d_{i}$, $d_{j}$ are relatively small). Therefore, ${\mathscr {Q}}$ measures the relative density of edges inside communities with respect to edges outside communities^26^.

For a non-directed weighted network ${\mathbb {G}}_{\text {weighted}}$, the modularity ${\mathscr {Q}}$ is defined almost the same as Eq. 11. However, for the weighted network, the adjacency matrix $A$ is replaced by the correlation matrix $C$, with matrix element $C _{ij}$ indicating the strength of the connection between node *i* and *j*. Correspondingly, $d_{i}$ and $d_{j}$ in the weighted network case are the sum of the weights of the edges connected to nodes *i* and *j*, and $m = \sum _{ij} C _{ij}$^27^.

For a directed weighted network ${\mathbb {G}}_{\text {directed}}$, the modularity ${\mathscr {Q}}$ is defined in Eq. (12):

12 $$\begin{aligned} {\mathscr {Q}} = \quad \frac{1}{2m}\sum _{ij}\left[ C _{ij}-\frac{d_{i}^{in}d_{j}^{out}}{2m}\right] \delta {(c_{i},c_{j})} \end{aligned}$$

where $d_{i}^{in}$ is the in-degree and $d_{i}^{out}$ is the out-degree of node *i*. The same intuition as for the non-directed network still applies in this case: to maximize ${\mathscr {Q}}$, we would assign node *i* and *j* to the same community (so delta function is 1) when the term $C_{ij}-\frac{d_{i}^{in}d_{j}^{out}}{2m}$ is relatively large. This term is large when there exists a strong edge from node *j* to *i* ($C_{ij}$ is large), while node *i* does not already have a large in-degree and node *j* does not already have a large out-degree ($d_{i}^{in}$ and $d_{j}^{out}$ are relatively small)^27^.

### Defining interbank network modularity

The interbank network in our work can be thought of as a directed weighted graph ${\mathbb {G}}_{\text {interbank}}$. The strength of edges are defined by the cross-holding matrix $C$, for instance, if bank *i* holds 20% of bank *j*’s shares, the $C _{ij} = 0.2$ and there is a directed edge pointing from node *j* to *i* in the graph ${\mathbb {G}}_{interbank}$ as edge direction specifies the direction of loss propagation. Under the systemic risk computational environment defined in section "Computational systemic risk environment", the network community in the interbank network would mean that banks within the same community share a large part of each other’s discontinuous value losses due to bank failures. The definition of modularity for ${\mathbb {G}}_{\text {interbank}}$ is similar to the modularity for ${\mathbb {G}}_{\text {directed}}$ defined in Eq. (12), and the in-degree of node *i* here corresponds to the losses that can be received by bank *i*, and similarly, the out-degree of node *j* corresponds to the losses bank *j* can cause in the network. *m* now is the total losses that can be incurred by all the banks in the network. Again the same intuition applies in our interbank network: we will assign bank *i* and bank *j* to the same community if the term $C _{ij}-\frac{d_{i}^{in}d_{j}^{\text {out}}}{2m}$ is relatively large. This term is large if bank *i* is vulnerable to the loss incurred by bank *j*’s failure ($C _{ij}$ is large), while bank *j* can not cause a very high loss in the network ($d_{j}^{\text {out}}$ is not high) and bank *i* can not incur a very high loss from other banks in the network ($d_{i}^{in}$ is not high). Following this intuition, the modularity of the interbank network is defined in Eqs.  (13)–(16):

13 $$\begin{aligned} {\mathscr {Q}}^{\text {interbank}}= & {} \quad \frac{1}{2m}\sum _{ij}\left[ C _{ij}-\frac{d_{i}^{in}d_{j}^{out}}{2m}\right] \delta {(c_{i},c_{j})} \end{aligned}$$

14 $$\begin{aligned} d_{i}^{in}= & {} \quad \sum _j C _{ij}\gamma v_j \end{aligned}$$

15 $$\begin{aligned} d_{j}^{out}= & {} \quad \sum _i C _{ij}\gamma v_j \end{aligned}$$

16 $$\begin{aligned} m= & {} \quad \sum _{i,j} C _{ij}\gamma v_j \end{aligned}$$

where $\gamma =0.217$ as mentioned in section "Financial shock and cascade failures simulation".

### Ising formulation for network modularity optimization

Network partitioning can be formulated as a simulated annealing optimization problem, which in turn can be formulated as a quantum annealing problem. Quantum computers can efficiently solve such problems and will eventually be able to scale to larger problems. Reichardt and Bornholdt^28^ have shown that the modularity function can be expressed as the Hamiltonian of an Ising spin glass. This connection between modularity and Ising spin glass is important since it can be exploited to develop new physics-inspired algorithms that maximize modularity using simulated annealing and more recently quantum annealing thanks to the advance of quantum computer hardware. We developed a quantum partitioning algorithm for directed and weighted graphs, which takes into account realistic physical constraints of interbank networks. We chose to implement our network partitioning using modularity with quantum annealing in order to compare to classical partitioning.

#### Quantum annealing

Quantum annealing (QA) is a particular type of adiabatic quantum computation (AQC) that follows the adiabatic theorem in quantum mechanics^29^. Das and Chakrabarti^30^ developed a general quantum annealing framework to solve hard optimization problems through adiabatic reduction of the quantum fluctuations in physical systems. This work provides a framework for realizing analog quantum computation. A comprehensive and complete review of Adiabatic Quantum Computing (AQC) is presented in Albash and Lidar^31^. They reviewed the major theoretical developments in AQC, the algorithmic advances, and their limitations. They provided examples solving hard optimization problems and also showed how AQC evolved into a universal alternative approach to gate-based quantum computers. A more recent and succinct overview on Adiabatic quantum computing was recently published by Rajak et al.^32^ They provided analytical and numerical evidence that quantum annealing can yield a better solution compared to simulated or thermal annealing in several glassy systems. These results corroborate the claim that quantum tunnelling has an advantage over thermal fluctuation in overcoming barriers with local minima and thus getting the system equilibrated around its corresponding ground state. They also gave insights into the still unresolved problems in the AQC area. Yarkoni et al.^33^ is another recent review paper on quantum annealing for use in solving many combinatorial optimization problems known to be NP-hard. The paper lays out the fundamental motivation behind quantum annealing, exhibits the state-of-the-art software and hardware used in quantum processors, and exposes different applications. The authors identified limitations and showed potential benefits for both researchers and practitioners in the industry.

##### Theorem 1

(Adiabatic theorem) Let $s := t/T$, $s \in [0,1]$, and ${\mathscr {H}}$ be a Hermitian operator (e.g. Hamiltonian). If ${\mathscr {H}}(s)$ varies smoothly with s, then for arbitrarily large T, ${\mathscr {H}}(s)$ varies slowly with t. In other terms, gradually changing conditions allow the system to adapt its configuration, hence the probability density is modified by the process. This behavior allows for Adiabatic quantum computation used by D-Wave quantum annealing optimization system.

D-Wave is a quantum computing company specializing in AQC^34^. D-Wave’s latest quantum computer, the D-Wave Advantage, uses a quantum processing unit (QPU) consisting of more than 5000 qubits and 35000 couplers and operates at temperatures below 15 mK ($-273.135^\circ C$). Physically implemented superconducting loops, or qubits, are arranged in unit cells on the QPU. Couplers are used to establish entanglements between qubits by interconnecting unit cells into architectures known as the Chimera or the Pegasus graphs.

D-Wave machines use QA to find the lowest energy eigenvalue of the Ising Hamiltonian, ${\mathscr {H}}_\text {Ising}$, which represents the minimal energy state (global minima of the physical system):

17 $$\begin{aligned} {\mathscr {H}}_\text {Ising} = \underbrace{-\frac{\Xi (s)}{2} \bigg ( \sum _{i} \sigma ^{(i)}_x \bigg )}_{\text {Initial tunneling Hamiltonian}} + \underbrace{\frac{\Gamma (s)}{2} \bigg ( \sum _{i} h_i \sigma ^{(i)}_z + \sum _{i>j} J_{i,j} \sigma ^{(i)}_z \sigma ^{(j)}_z \bigg )}_{\text {Final problem Hamiltonian}} \end{aligned}$$

where:

- $\Xi (s)$ is the tunneling energy.
- $\Gamma (s)$ is the problem Hamiltonian energy
- $\sigma ^{(i)}_{x}$ and $\sigma ^{(i)}_{z}$ are the Pauli matrices acting on qubit *i*.
- $h_i$ is the qubit bias.
- $J_{i,j}$ is the coupling strength between qubits *i* and *j*.

At the beginning of the annealing process, the system is in the ground state which corresponds to the lowest energy eigenstate of the tunneling Hamiltonian. At the end of the annealing process, the system’s energy eigenstate is that of the final problem Hamiltonian. The adiabatic theorem states that if the annealing process is done successfully, the system remains in the ground state throughout the process, which corresponds to the global minimum from an optimization perspective.

#### Ising formulation for network modularity

For a problem to be solved by D-Wave, it has to be mapped onto an Ising or Quadratic unconstrained binary optimization (QUBO) objective function, which are respectively defined by Calude et al.^35^ as:

18 $$\begin{aligned} {\mathscr {E}}_\text {Ising}(s) = \sum _{i=1}^{N} h_i s_i + \sum _{i=1}^{N} \sum _{j=i+1}^{N} J_{i,j} s_i s_j \qquad \text {(Ising)} \end{aligned}$$

19 $$\begin{aligned} f(x)=\sum _{i} Q_{i,i} x_i + \sum _{i<j} Q_{i,j} x_i x_j =\underset{x \in \{0,1\}^n }{\text {min}} x^TQx \qquad \text {(QUBO)} \end{aligned}$$

where:

- $J_{i,j}$ and $h_i$ are the same parameters that appear in ${\mathscr {H}}_\text {Ising}$.
- *Q* is an upper-triangular $N \times N$ matrix of real coefficients which serve as weights.
- $$s \in \{-1, 1\}$$ and $$x \in \{0, 1\}$$.

We can easily go from the Ising formulation to the QUBO formulation by using the following change of variable $s=2x-1$.

The optimization of ${\mathscr {Q}}$ in Eq. (13) can be reformulated using a Binary Quadratic Model (BQM), which can encode Ising and QUBO models and can be solved using a heuristic such as Classical Annealing or Quantum Annealing^36^. They use a matrix of binary variables $X$ with size $n \times k$ with *n* being the number of nodes and *k* being the number of communities. Each element $x_{i,j}$ indicates whether node *i* is in community *j*. Node *i* is in community *j* if $x_{i,j} = 1$ and 0 otherwise. As each node only belongs to a single community, each node *i* must satisfy:

20 $$\begin{aligned} \sum _{j=1}^{k} x_{i,j} = 1 \quad \forall i \end{aligned}$$

We define $x_{j}$ as the jth column vector for matrix $X$ and we define matrix $B$ in Eq. (21):

21 $$\begin{aligned} B _{i,j} = C _{i,j} - \dfrac{d_{i}d_{j}}{2m} \end{aligned}$$

The modularity ${\mathscr {Q}}$ can be written as:

22 $$\begin{aligned} \mathscr {Q}({X}) = \sum _{j=1}^{k} (x_{j}^T B x_{j}) \end{aligned}$$

The matrix $X$ generalizes the $\delta {(c_{i},c_{j})}$ in Eq. (11), which enables us to find multiple communities within a network. The optimal community partition of the network corresponds to the maximum ${\mathscr {Q}}$ value.

#### Ising formulation for interbank network modularity

The network community partitioning problem described in section "Defining network modularity" can be formulated as a constrained integer linear programming problem and solved using Quantum Annealing. Quantum Annealing can be implemented using the open source D-wave system^37^. In this section, we first discuss in detail the general framework for using Quantum Annealing to solve a constrained integer linear programming problem, then we derive a new BQM formulation of interbank network modularity (${\mathscr {Q}}^{\text {interbank}}$ as defined in Eqs. (13)–(16)) as a special case of this framework.

Let us consider the following integer linear programming problem in the most general form:

23 $$\begin{aligned} \underset{z}{\min }\,\hat{{\mathscr {H}}}(z) \end{aligned}$$

subject to *k* constraints:

24 $$\begin{aligned} A_{\sigma }z=\lambda _{\sigma } \quad \sigma =0,1,\dots , k-1 \end{aligned}$$

$\hat{{\mathscr {H}}}(z)$ is the cost function that we are trying to minimize. Each $A_{\sigma }$ is a matrix of dimension $n_{\sigma }\times 4N^2$ and $\lambda _{\sigma }$ are vectors of length $n_{\sigma }$.

The vector *z* contains the optimization variables and we restrict ourselves to considering only the case where all *z* are positive integers. The cost function $\hat{{\mathscr {H}}}(z)$ is the linear part of the Hamiltonian. For the constraints, we need to convert them into quadratic Hamiltonians. For a constraint $A_{\sigma }z=\lambda _{\sigma }$, we can derive a quadratic cost function as follows:

25 $$\begin{aligned} \begin{aligned} (A_{\sigma }z - \lambda _{\sigma })^2&= (A_{\sigma }z - \lambda _{\sigma })^T(A_{\sigma }z - \lambda _{\sigma }) \\&= (A_{\sigma }z)^T(A_{\sigma }z) - \lambda _{\sigma }^T(A_{\sigma }z)-(A_{\sigma }z)^T\lambda _{\sigma } + \lambda _{\sigma }^T\lambda _{\sigma } \\&= z^T(A_{\sigma }^TA_{\sigma })z - (\lambda _{\sigma }^TA_{\sigma })z - z^T(A_{\sigma }^T\lambda _{\sigma }) + \lambda _{\sigma }^T\lambda _{\sigma } \end{aligned} \end{aligned}$$

Hence, the total cost (Hamiltonian) is:

26 $$\begin{aligned} \text {cost}= \hat{{\mathscr {H}}}(z) + \sum _{\sigma =0}^{k-1}\Big [ z^T(A_{\sigma }^TA_{\sigma })z - (\lambda _{\sigma }^TA_{\sigma })z - z^T(A_{\sigma }^T\lambda _{\sigma }) + \lambda _{\sigma }^T\lambda _{\sigma } \Big ] \end{aligned}$$

Since $\lambda _{\sigma }^T\lambda _{\sigma }$ is a constant, we can drop it from the cost. To make the cost more general we can also add weights to various parts of it, instead of just summing the minimization cost and the constraints. Let us add a weight *w* in front of the cost function $\hat{{\mathscr {H}}}(z)$, so that we can better control what is more important, the minimization of the cost, or the constraints (large *w* will emphasize the role of the minimization of the cost, whereas small *w* will emphasize the role of the constraint). So for the final cost function we have:

27 $$\begin{aligned} \text {cost} = w \hat{{\mathscr {H}}}(z) + \sum _{\sigma =0}^{k-1}\Big [ z^T(A_{\sigma }^TA_{\sigma })z - (\lambda _{\sigma }^TA_{\sigma })z - z^T(A_{\sigma }^T\lambda _{\sigma })\Big ] \end{aligned}$$

For further convenience, we write the cost by explicitly expanding the matrix multiplications using indices:

28 $$\begin{aligned} \begin{aligned} \text {cost}&= w \hat{{\mathscr {H}}}(z) + \sum _{\sigma =0}^{k-1}\left[ \sum _{\mu \nu }(A_{\sigma }^TA_{\sigma })_{\mu \nu }z_{\mu }z_{\nu } - \sum _{\mu }(\lambda _{\sigma }^TA_{\sigma })_{\mu }z_{\mu } - \sum _{\mu }(A_{\sigma }^T\lambda _{\sigma })_{\mu }z_{\mu }\right] \\&=w \hat{{\mathscr {H}}}(z) + \sum _{\sigma =0}^{k-1}\left[ \sum _{\mu \nu }(A_{\sigma }^TA_{\sigma })_{\mu \nu }z_{\mu }z_{\nu } - \sum _{\mu }(\lambda _{\sigma }^TA_{\sigma }+ A_{\sigma }^T\lambda _{\sigma })_{\mu }z_{\mu }\right] \end{aligned} \end{aligned}$$

We know that the first $2N^2$ variables in *z* are positive non-binary integers and the last $2N^2$ variables are binary integers. We can generalize this by assuming that the number of non-binary integers is given to us via a parameter *M*. Note that we still assume that the *M* non-binary variables are queued in the beginning of the vector *z* and the rest are the binary variables.

A non-binary positive integer can be written in a binary expanded form using *D* bits, as follows:

29 $$\begin{aligned} z_{\mu } = \sum _{i=0}^{D-1}2^{i}x_{\mu ,i} \quad \mu =0,1,\dots ,2N^2-1 \end{aligned}$$

Here $x_{\mu ,i}$ are the bits in the binary representation of $z_{\mu }$. After expanding all non-binary integers in terms of *D* bits, instead of the $4N^2$ variables $z_{\mu }$, we will have $4N^2 + M (D-1)$ binary variables in our problem, and we can encode it into a D-Wave machine.

In order to implement the cost function in terms of binary variables $x_{\mu ,i}, \mu =0,\dots ,2N^2-1, i=0,\dots ,D-1$ and $z_{\mu }, \mu =2N^2-1,\dots ,4N^2-1$, note that:

30 $$\begin{aligned} z_{\mu }z_{\nu } = \sum _{i=0}^{D-1}2^{i}x_{\mu ,i}\sum _{j=0}^{D-1}2^{j}x_{\nu ,j} = \sum _{i,j=0}^{D-1}2^{i+j}x_{\mu ,i}x_{\nu ,j} \end{aligned}$$

In the case $\mu =\nu$, it can be further simplified as:

31 $$\begin{aligned} z_{\mu }^2 = \left( \sum _{i=0}^{D-1}2^{i}x_{\mu ,i} \right) ^2 = \sum _{i,j=0}^{D-1}2^{i}x_{\mu ,i}2^{j}x_{\mu ,j} = \sum _{i,j=0}^{D-1}2^{i+j}x_{\mu ,i}x_{\mu ,j} = \sum _{i=0}^{D-1}4^ix_{\mu ,i} + \sum _{i \ne j}^{D-1}2^{i+j}x_{\mu ,i}x_{\mu ,j} \end{aligned}$$

where we have used the fact that the square of a binary variable is equal to itself ($x_{\mu ,i}^2=x_{\mu ,i}$). Thus, the final form of the cost function containing only binary variables is as follows:

32 $$\begin{aligned} \begin{aligned} cost = w \hat{{\mathscr {H}}}(z)&+ \sum _{\sigma =0}^{k-1} \bigg [ \sum _{\mu \nu =0}^{M-1}(A_{\sigma }^TA_{\sigma })_{\mu \nu }\sum _{i,j=0}^{D-1}2^{i+j}x_{\mu ,i}x_{\nu ,j} + \sum _{\mu \nu =M}^{4N^2-1}(A_{\sigma }^TA_{\sigma })_{\mu \nu }z_{\mu }z_{\nu } \\&+ \sum _{\mu =M, \nu =0}^{4N^2-1,M-1}(A_{\sigma }^TA_{\sigma })_{\mu \nu }z_{\mu }\sum _{i=0}^{D-1}2^{i}x_{\nu ,i} + \sum _{\mu =0, \nu =M}^{M-1,4N^2-1}(A_{\sigma }^TA_{\sigma })_{\mu \nu }\sum _{i=0}^{D-1}2^{i}x_{\mu ,i}z_{\nu } \\&- \sum _{\mu =0}^{M-1}(\lambda _{\sigma }^TA_{\sigma } + A_{\sigma }^T\lambda _{\sigma })_{\mu }\sum _{i=0}^{D-1}2^{i}x_{\mu ,i} - \sum _{\mu =M}^{4N^2-1}(\lambda _{\sigma }^TA_{\sigma }+ A_{\sigma }^T\lambda _{\sigma })_{\mu }z_{\mu } \bigg ] \end{aligned} \end{aligned}$$

This formulation of the constrained integer linear programming problem can be solved in the D-Wave Quantum Annealer.

The interbank network community partitioning problem discussed in section "Defining interbank network modularity", and more specifically, the constrained optimization problem defined in Eqs. (14) and (12) can be viewed as a special and simpler case of the Hamiltonian (32). The special case corresponds to the situation where the number of non-binary integers $M=0$ and the number of bits in the binary expansion $D=1$ in Eq. (29).

Again, the modularity optimization problem with $x_{i,j}$ being a binary variable can be written as follows:

33 $$\begin{aligned} \begin{aligned}{}&\max _x{} & {} \sum _{j=1}^{k} (x_{j}^T B x_{j}) \\&\text {subject to}{} & {} \sum _{j=1}^{k} x_{i,j} = 1 \quad \forall i. \end{aligned} \end{aligned}$$

Using a similar method as in Eq. (20), we can fold the constraints into the objective function and get a quadratic cost function form with $\lambda _{i}$ being the corresponding relaxation coefficients, and assign weight $w$ to the objective function. When we also expand the vector $x_{j}$ into variables $x_{i,j}$, the optimization becomes:

34 $$\begin{aligned} \max _x w\sum _{j=1}^{k} \sum _{i=1}^{n} \sum _{m=1}^{n} B _{im}x_{i,j}x_{m,j} + \sum _{i=1}^{n} \lambda _{i} \left( \sum _{j=1}^{k} x_{i,j} - 1\right) ^2 \end{aligned}$$

which can be further expanded into:

35 $$\begin{aligned} \max _x w\sum _{j=1}^{k} \sum _{i=1}^{n} \sum _{m=1}^{n} B _{im}x_{i,j}x_{m,j} + \sum _{i=1}^{n} \lambda _{i} \left( \sum _{j=1}^{k} \sum _{l=1}^{k} x_{i,j}x_{i,l} - 2 \sum _{j=1}^{k} x_{i,j} + 1\right) \end{aligned}$$

where we have used the fact that the square of a binary variable is equal to itself. The first term in Eq. (35) corresponds to the term $w\hat{{\mathscr {H}}}(z)$ in Eq. (28). Now all the decision variables $X$ are directly binary instead of integers *Z* and this Hamiltonian $\hat{{\mathscr {H}}}(z)$ is already in the quadratic form. The second term represents the Lagrange multipliers used to encode constraints from Eq. (33).

### Two-stage optimization algorithm

This section discusses our two-stage optimization algorithm which combines the one-stage optimization model from Section "Mixed integer linear programming formulation" with the network partitioning algorithms from sections "Defining interbank network modularity" and "Ising formulation for network modularity optimization". More specifically, we first apply network community partitioning to the interbank network to find highly connected communities (modules) of banks, then we apply the one-stage optimization to individual modules, which themselves are smaller interbank networks. The algorithm is shown in Algorithm 2.
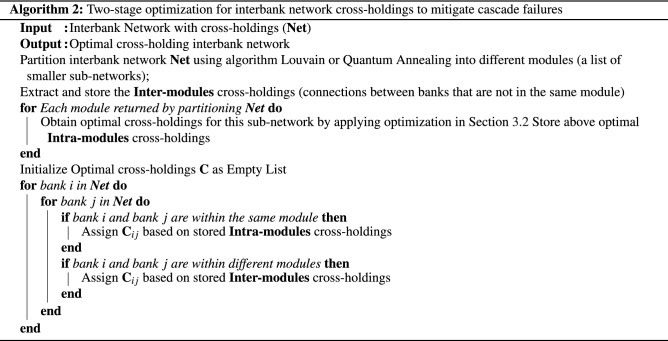


There are two stages in Algorithm 2. The first stage is network partitioning of the interbank network using algorithms discussed in sections "Defining interbank network modularity" and "Ising formulation for network modularity optimization". One thing to notice is that we need to keep a record of all the cross-holdings between banks from different modules (inter-module). These are the cross-holdings that we are not optimizing in the second stage, and we need to use them to reconnect the optimized modules. Intuitively, these cross-holdings that are not included in our optimizations correspond to the liability connections that are not really contributing to the cascade failures because modularity guarantees weaker links between modules compared to those within modules. For instance, as discussed in section "Motivation for using network modularity", cascade failures generally develop within each module at the early iterations of cascade simulation, so these left-out cross-holdings are the cross-holdings that only convey the bank failures at later stages of the cascade when the cascade failures have already gone out of control, therefore, there is no benefit to optimizing these cross-holdings.

**Figure 7:**
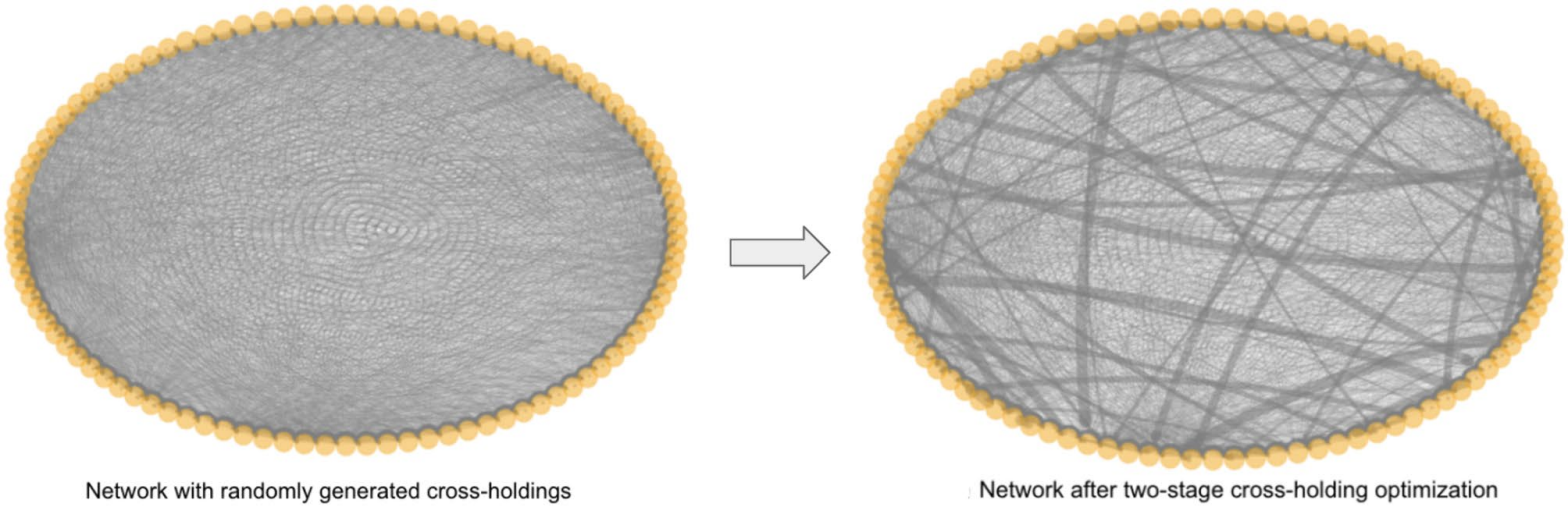
Example interbank network using two-stage optimization. The left plot is the circular layout visualization of a randomly generated interbank network shown in Fig. 4 with 100 banks. The right plot is the network after two-stage optimization using Algorithm 2. Notice that the change in cross-holdings is not as drastic as in Fig. 4.

The second stage is individual module optimization using the same MILP optimization algorithm (one-stage optimization) developed in section "Mixed integer linear programming formulation". Again within each module, we are minimizing the total incurred discontinuous losses due to potential failures of all banks and we achieve this minimization by finding the optimal cross-holdings $C _{ij}$ for all bank *i* and bank *j*. Figure 7 is one visualization of how such optimization changes the cross-holding structure of a randomly generated example interbank network shown in circular layout.

**Figure 8:**
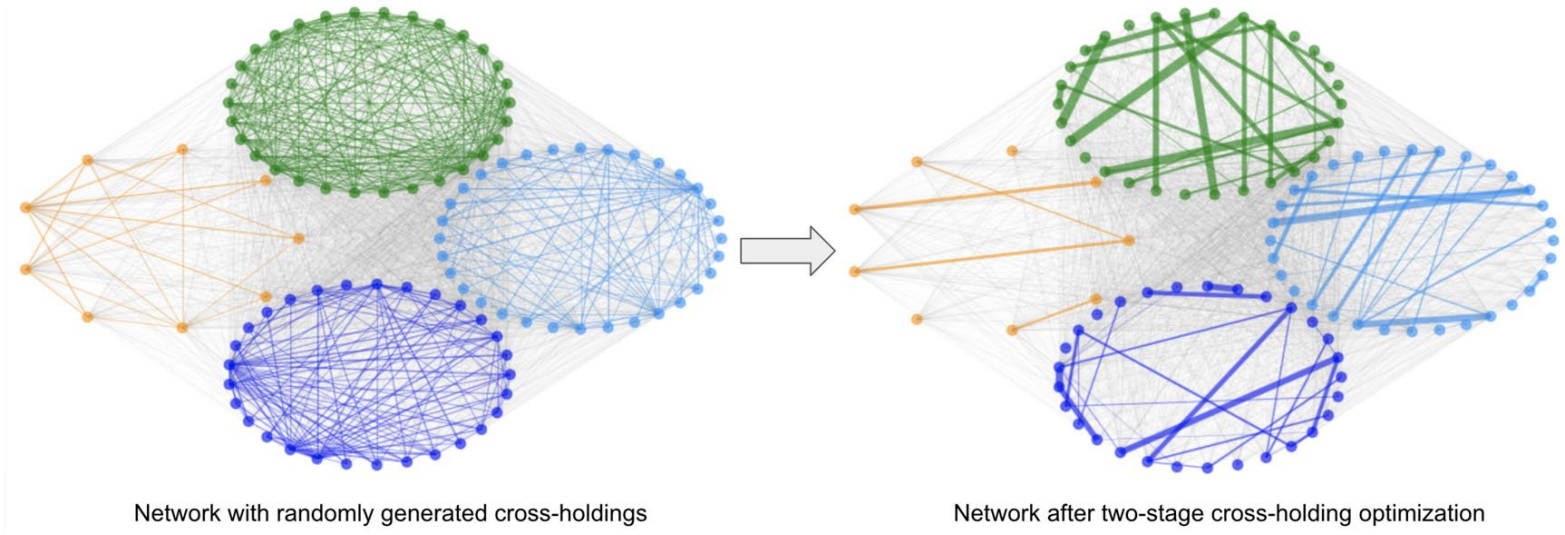
Two-stage optimization visualization using the same example network as in Fig. 7 showing communities in different colors using a circular layout. We can see clearly that the cross-holdings (edges) within the four communities are individually optimized and the cross-holdings (edges) between different communities are unchanged.

The network shown in Fig. 7 is the same interbank network shown in Fig. 4. Similar to the one-stage optimization visualization in Fig. 4, there is a contraction of cross-holdings during the optimization from the random generated network. One thing to notice is that the concern raised at the end of section "Mixed integer linear programming formulation" about the large change in the cross-holdings is, to some extent, mitigated by the two-stage optimization where the change of cross-holdings is not as drastic as in the one-stage optimization in Fig. 4. This reduction in the changes of the cross-holdings results in a solution that is more realistic for implementation by the regulators. In the two-stage optimization algorithm, the optimizations are only operating within each module and thus the changes in cross-holdings are more local in scale, which is also more realistic. Figure 8 shows the visualization of the two-stage optimization for the same network in Fig. 7 while showing modules in different colors. The four modules are individually optimized while the connections between banks in different modules (grey lines) are unchanged.

## Performance and scalability

### Performance and scalability of two-stage with classical partitioning

**Figure 9:**
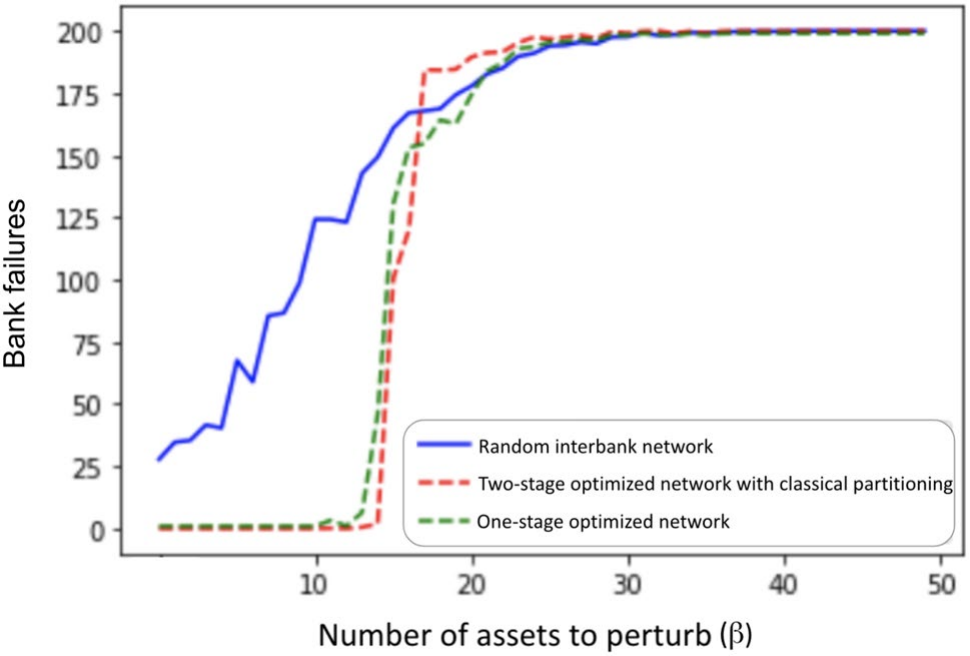
Average cascade simulation results showing bank failures relative to an increasing number of perturbed assets ($\beta$) for random network, one-stage optimized network, and two-stage optimized network. For each $\beta$, 10 random interbank networks are generated. We generate an asset perturbation to start the cascade, record the total number of bank failures after the cascade simulation terminates, take the average of number of failures of the 10 networks for each optimization, and plot the average numbers on the y-axis for the given $\beta$.

We tested the performance of the interbank network optimizations algorithms described in previous sections. The experiment used here is the same one shown in Fig. 3, in which we vary the number of perturbed assets $(\beta )$ input to cascade simulation (Algorithm 1), and plot the total number of failed banks after the simulation terminates. Ideally, we want to postpone the cascade or phase transition shown in Fig. 3 to larger $\beta$ values. The cascade simulation results for random unoptimized networks and the associated one-stage and two-stage optimized networks are shown in Fig. 9. At each $\beta$ value, we generate 10 random networks and for each random network we apply two different optimizations (one-stage and two-stage). Therefore for each $\beta$ we have ten sets of networks with each set containing one random network, one optimal network from the one-stage optimization, and one optimal network from the two-stage optimization, defined as Random, One-stage optimal, and Two-stage optimal. The final number of failures after convergence at each $\beta$ shown in Fig. 9 is the average result of 10 runs of each set.

In Fig. 9, we can see that both one-stage optimization and two-stage optimization can postpone the phase transition to larger $\beta$ values. The rightward shift of the phase transition from $\beta$ around 5 to around 14 indicates that the optimal interbank network is more resilient to perturbation, and can withstand more intense asset perturbation without exhibiting cascade failures. We see similar performance from the one-stage optimization and the two-stage optimization in Fig. 9 in terms of mitigating cascade failures.

Two-stage optimization has three advantages over one-stage optimization: First, the two-stage optimization algorithm requires less change in the cross-holding structures to achieve similar cascade failure mitigation, which is more realistic and achievable by banks in real life. Second, as shown in Fig. 9, two-stage optimization slightly outperformed one-stage optimization, and two-stage withstands two additional perturbed assets before exhibiting cascade failures. Third and most importantly, the computation for two-stage optimization is far more efficient than the one-stage optimization. As discussed in section "The curse of dimensionality", solving the MILP for the whole network in one-stage optimization becomes intractable when the size of the network is large, however, two-stage optimization successfully reduces the size of the optimization problem by partitioning the problem into multiple smaller networks.

**Figure 10:**
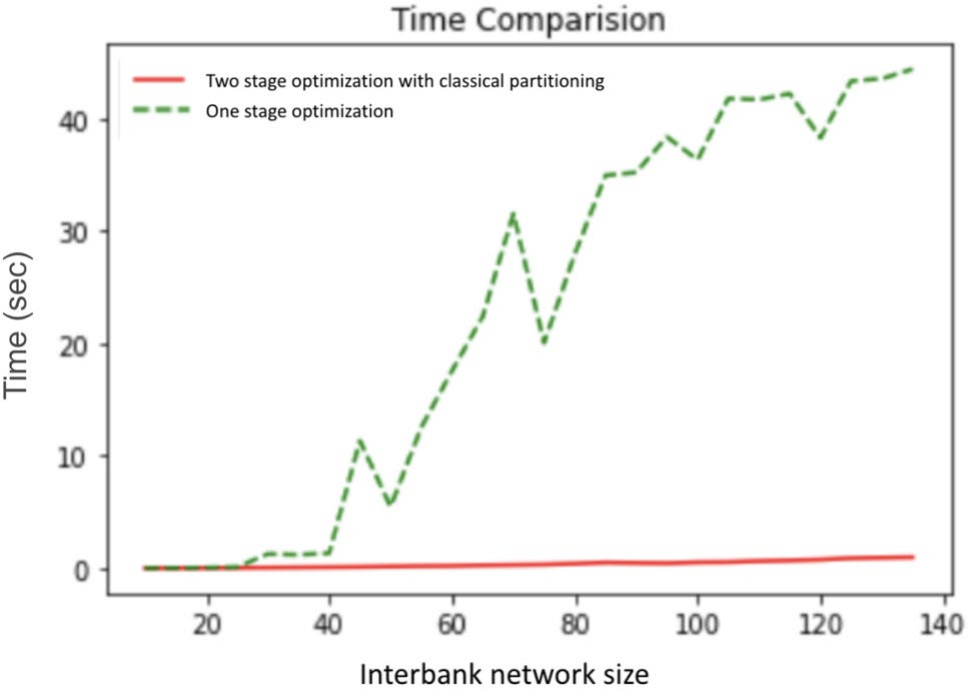
Time comparison of one-stage optimization and two-stage optimization for increasing interbank network size. The x-axis shows the size of the interbank network that is being optimized. The y-axis shows the total running time for one-stage optimization and two-stage optimization. We see a considerable running time improvement using two-stage optimization compared to one-stage optimization.

The time complexity comparison between the two algorithms is shown in Fig. 10. We vary the number of banks when generating the interbank network and then at each network size we take the average of the computation times for optimizing ten networks using the two algorithms. We can see that, apart from noisiness from random generation, there is rapid growth in the computation time of one-stage optimization, while the computation time for two-stage optimization grows linearly. We control the number of modules so that the number of banks per module does not change as the interbank network size increases. Therefore the size of individual optimizations at the second stage will be approximately the same. In other words, it is only the number of optimizations that is increasing rather than the size of each individual optimization problem. The time complexity is linear because the number of modules grows linearly with the number of banks. Therefore, two-stage optimization can drastically reduce the time complexity of the interbank network optimization, easily scale to more realistic interbank network with larger sizes, while keeping a similar or even better performance than the one-stage optimization.

### Quantum computing implementation

**Figure 11:**
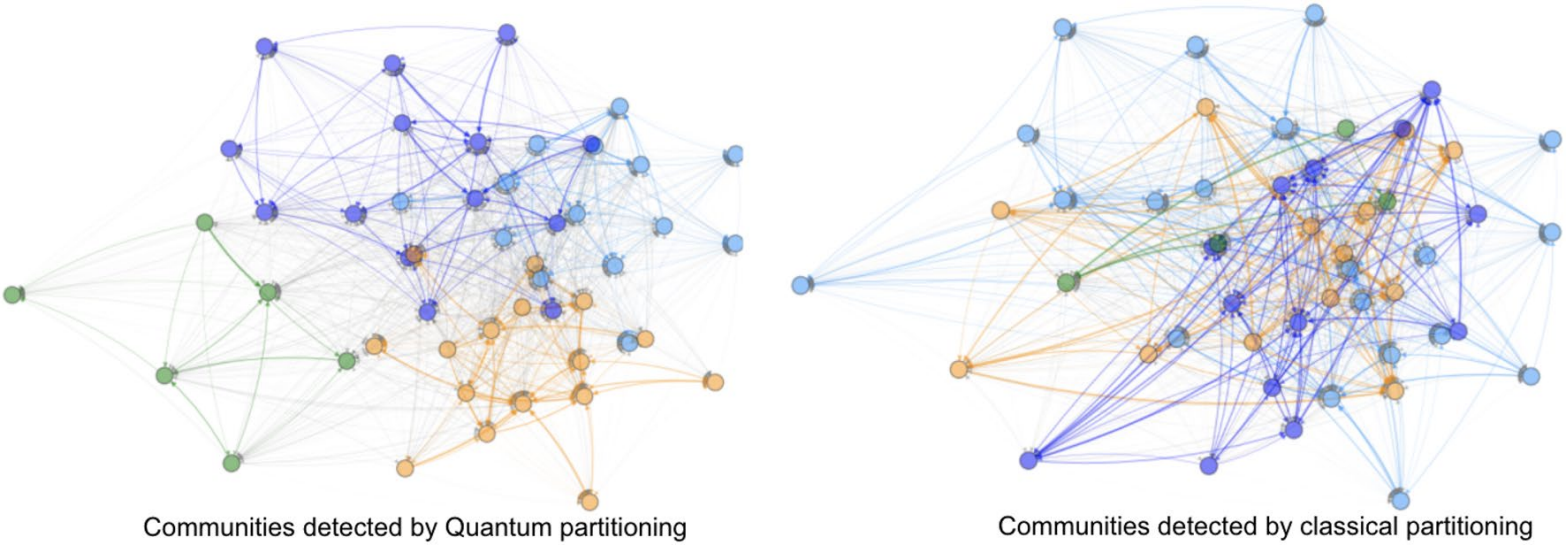
Comparison of quantum and classical partitioning for an example interbank network.

In this section, we discuss some of the challenges we encountered during the implementation of the two-stage optimization method (Algorithm 2) with quantum partitioning (discussed in section "Ising formulation for network modularity optimization"). As a reminder, in our two-stage optimization, we first partition the interbank network into different modules based on the cross-holdings, then we optimize each module individually. By adapting quantum partitioning discussed in section "Defining interbank network modularity", we hope to find better interbank network modules (Fig. 11) with reduced computational time, thus optimizing more important connections and achieving better cascade mitigation when compared to classical heuristic partitioning.

The Ising formulation of the interbank partitioning problem in Eq. (35) is discussed in detail in section "Defining interbank network modularity". We worked with two D-wave systems to solve this particular quantum annealing (QA) problem. We initially used the D-wave 2000Q solver in a D-wave system with 2048 qubits and Chimera graph embedding^34^. We upgraded to using the D-Wave Advantage System 1.1 5000Q solver in a D-wave system with 5000 qubits and a different topology with Pegasus (P16) graph embedding. We terminate our quantum partitioning algorithm when it iterates three times without a change in interbank modularity.

**Figure 12:**
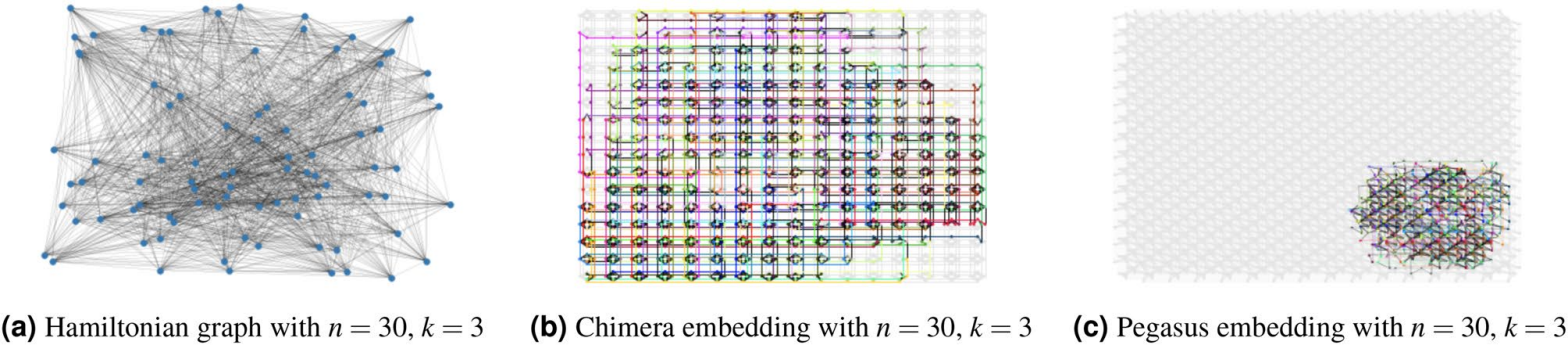
Example of spin Hamiltonian graph with Chimera and Pegasus embeddings $(n=30)$ and $(k=3)$.

Figure 12 shows the process of implementing QA for binary optimization problems. In this example, we partition a network of 30 banks $(n=30)$ into 3 modules $(k=3)$ due to quantum hardware constraints, therefore, following Eq. (35), we would have a binary quadratic model (BQM) problem with 90 binary decision variables. This BQM problem corresponds to a Hamiltonian of a spin system visualized as a graph shown in Fig. 12a with 90 nodes. In the spin Hamiltonian graph, each bank expands into 3 spin particles indicating which modules the bank is in. For any spin Hamiltonian system, D-Wave conducts topological embedding to map the partitioning optimization problem into a D-Wave hardware compatible form using different quantum processing unit (QPU) topologies. One such embedding is the minor embedding using a Chimera or a Pegasus embedding graph^38^. Figure 12b is the Chimera graph embedding of the particular spin Hamiltonian system shown in Fig. 12a, while Fig. 12c is the Pegasus graph embedding. We can see that the Pegasus graph embedding enabled by upgraded 5000-qubit hardware has higher embedding capacity compared to the Chimera graph embedding, therefore we used it for our experiments.

**Figure 13:**
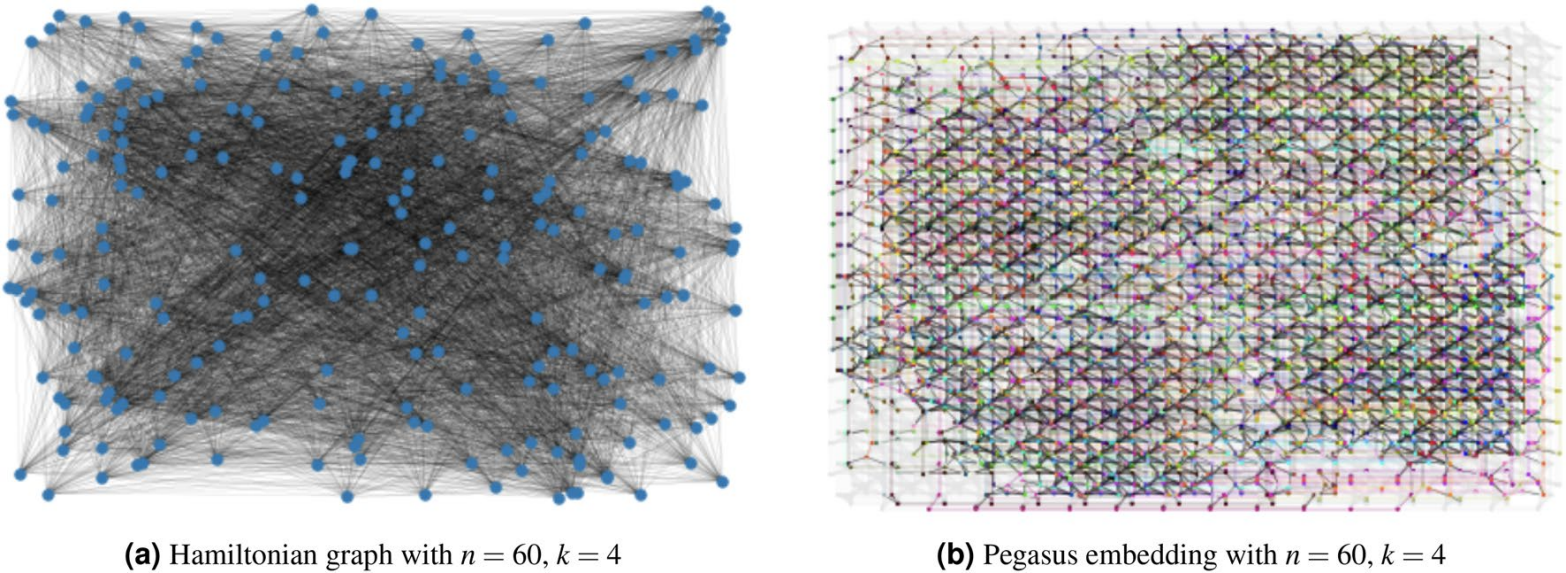
Example of spin Hamiltonian graph with Chimera and Pegasus embeddings $(n=60)$ and $(k=4)$.

**Figure 14:**
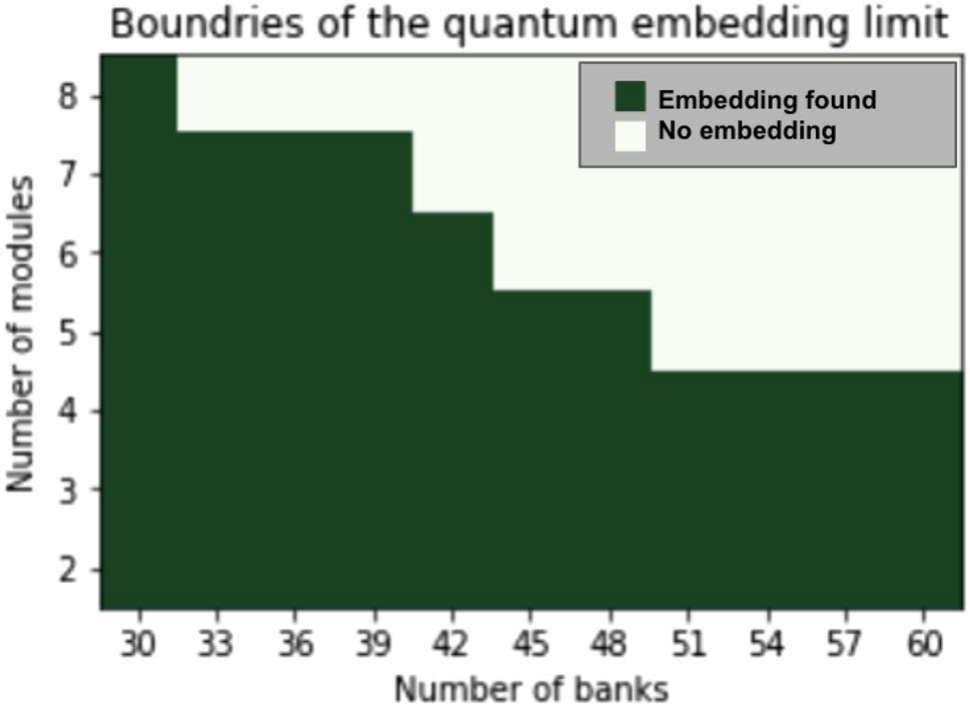
Set of possible quantum interbank network embeddings.

However, due to the hardware limitations and the use of this minor embedding, there is a limitation on the size of the interbank network partitioning problem that can be solved using QA. Figure 13a shows a spin Hamiltonian graph with $(n=60)$ and $(k=4)$ while Fig. 13b shows the Pegasus embedding with almost all of the embedding space utilized. Figure 14 shows the boundary of the existence of an embedding for a varying number of banks (*n*) and modules (*k*). This embedding boundary limits the scalability of our two-stage optimization with quantum partitioning, therefore, in section "Performance and scalability of two-stage with quantum partitioning" when we compare the algorithms’ performance, we limit the size of the interbank network to be $(n=50)$ to ensure there is always an embedding. When $n=50$, *k* generally is smaller than 6, and $n=50$, $k=6$ is inside the boundary. If $k\ge 6$ for a particular randomly generated interbank network, we discard that network and generate a new one. This upgrade from a 2000-qubit system to a 5000-qubit system shows how quantum computing will scale as the hardware improves.

### Performance and scalability of two-stage with quantum partitioning

**Figure 15:**
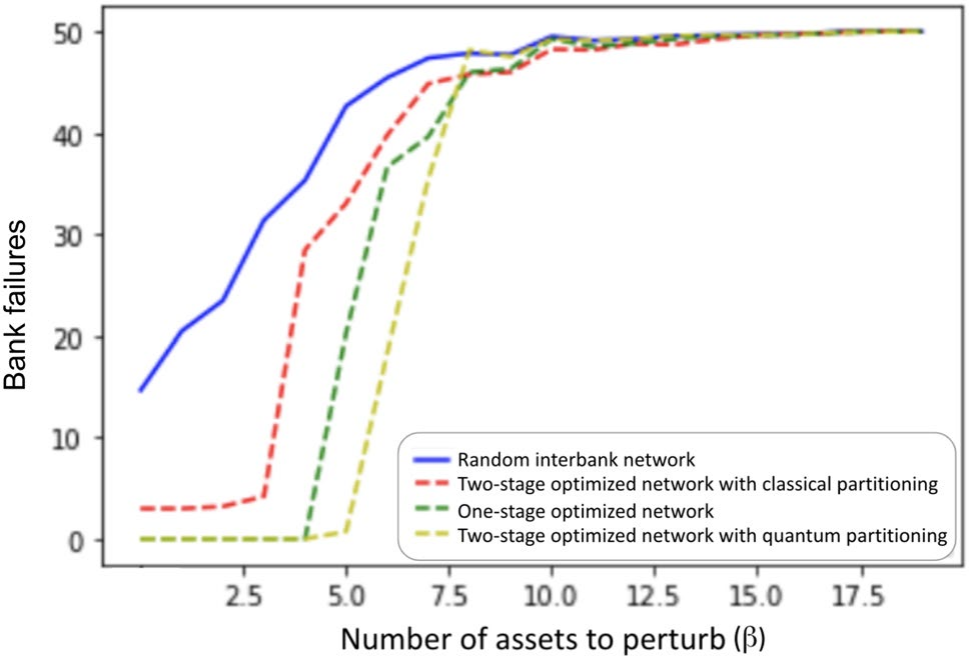
Cascade failure simulation results for random network, one-stage optimization, and classical and quantum two-stage optimization for an interbank network of 50 banks.

**Figure 16:**
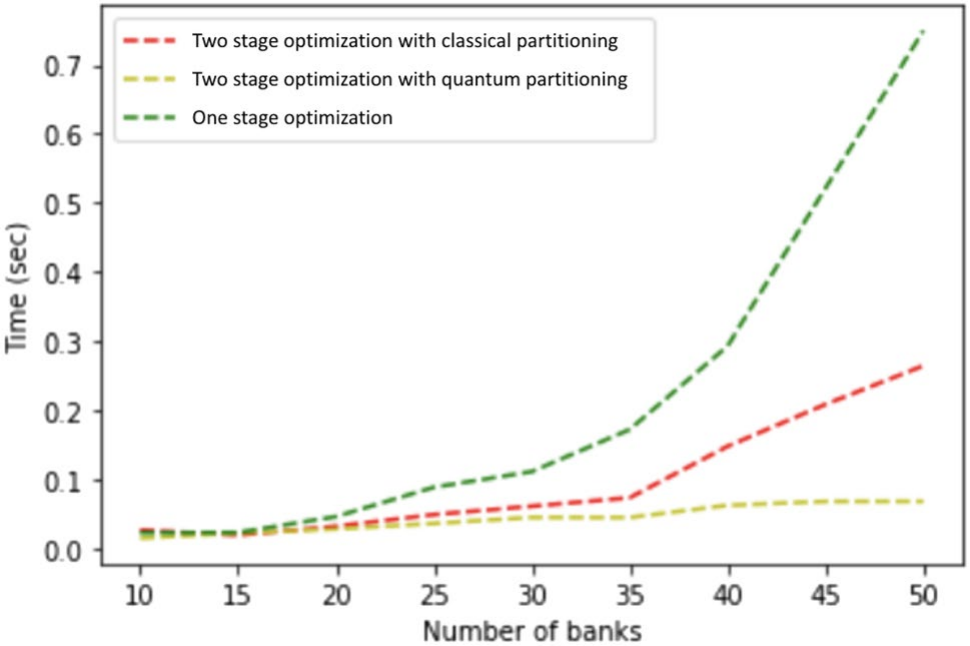
Time comparison of one-stage optimization and classical and quantum two-stage optimization for an interbank network of 50 banks. Two-stage optimization with quantum partitioning takes much less time.

**Figure 17:**
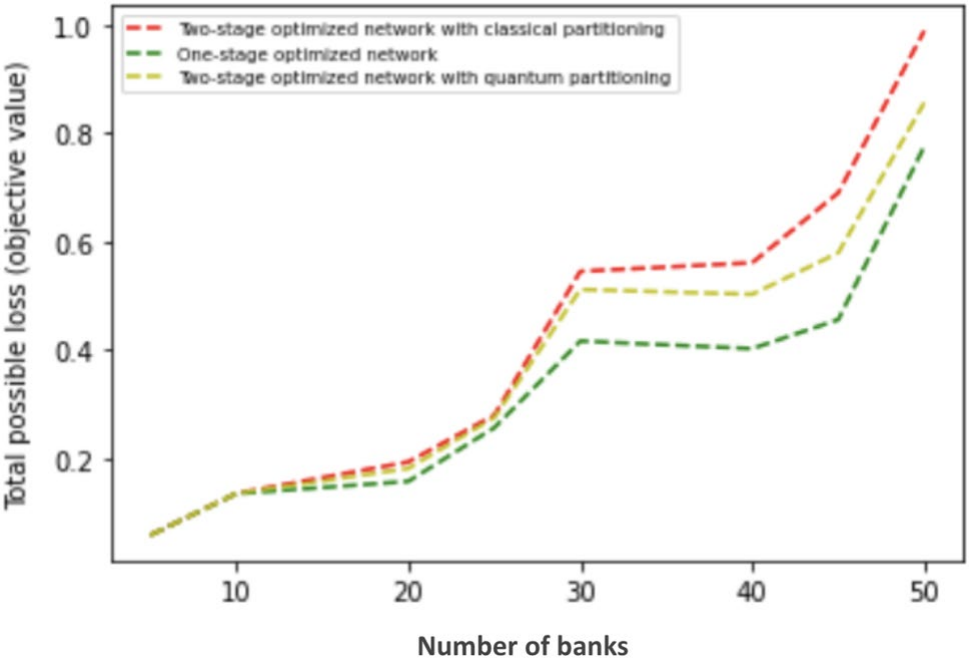
One stage optimization has a slight advantage over two-stage optimization when measured by total possible loss. However, one-stage optimization underperforms two-stage optimization in terms of delaying cascade failures as shown in Fig. 15.

We tested the performance of two-stage optimization with quantum partitioning. With the D-Wave system used for testing, we are constrained to a network at most 50 banks. Larger networks will be able to be handled by larger quantum computers in the future.

Figure 15 shows the same type of performance results as Fig. 9 with the addition of the quantum optimization approach. Notice that the unoptimized network starts off with 1/3 of the banks failing in the 50 bank network but only 1/8 of the banks failing in the 200 bank network. For the 50-banks network, the phase transition for the two-stage with classical partitioning does not quite match the one-stage optimization as it did with the 200-banks network.

Two-stage with quantum partitioning outperforms both of the classical methods. It beats the two-stage with classical partitioning because it is able to find better partitions that are able to mitigate cascades more effectively. It surprisingly beats the one-stage optimization, demonstrating that appropriate network partitioning actually results in better cross-holdings optimization than with global one-stage optimization, as with accurate network partitioning, two-stage optimization is optimizing more important cross-holdings and confining the source of the cascade more accurately.

Figure 16 shows the same type of time complexity results as Fig. 10 with the addition of the quantum optimization approach. It shows that the two-stage with classical partitioning time complexity increases more slowly than the one-stage, and quantum partitioning shows very little increase.

Figure 17 shows the total possible loss relative to an increasing number of banks. It also shows that two-stage optimization comes with higher total possible loss while outperforming one-stage optimization in terms of delaying cascade failures as shown in Fig. 15. Our conclusion is that having the minimum total possible loss does not mean having the best performance in terms of delaying cascade failures. Our results show that minimizing the total possible loss is not the best way to mitigate systemic risk and demonstrate that optimizing cross-holdings locally within each module better confines the contagion and hence better delays cascade failures.

### A heatmap visualization of cascade failures

**Figure 18:**
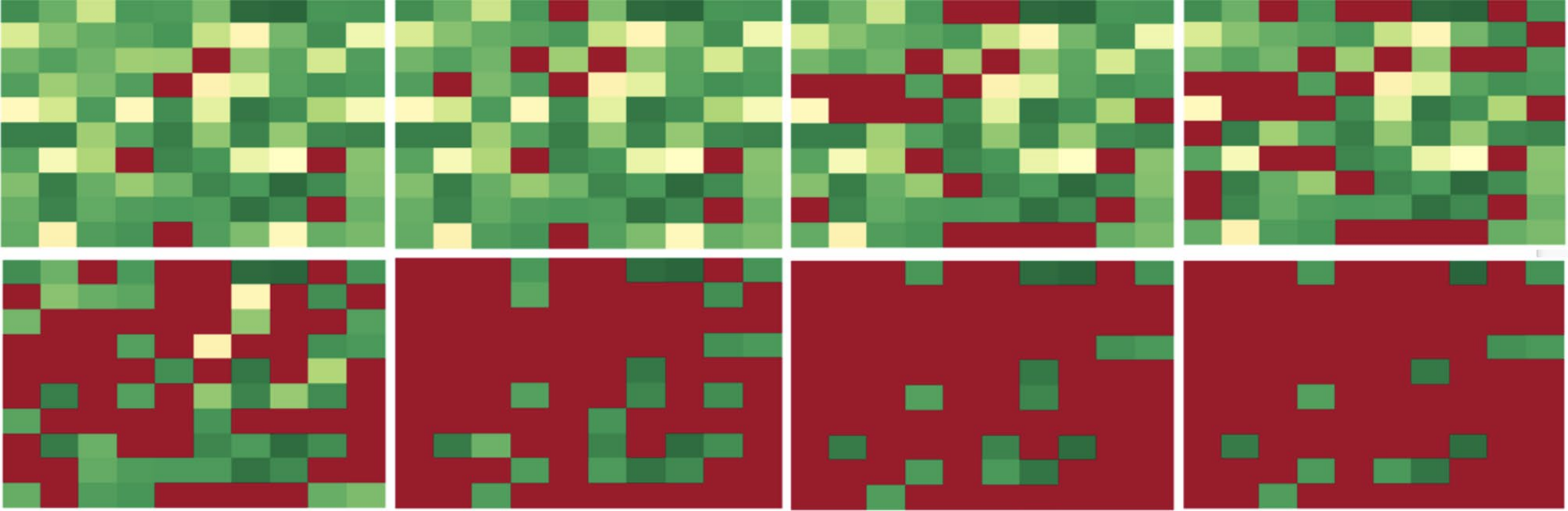
Heatmap visualization of cascade simulation for a random network.

For further insight into the cascade failure process, we developed a heatmap visualization tool. The heatmap displays the interbank network as an array of cells with each cell being a bank and the color of the cell indicating the relationship of that bank’s current value with its critical value $v_c$. If the bank’s value is higher than $v_c$, the corresponding cell has a color within a range from yellow to green, and the higher the value, the more green the cell. If the bank’s value is below $v_c$, the bank is failed and the color of corresponding cell is red. Banks are randomly assigned to cells without regard to connectivity. Figure 18 shows the cascade process of a random network with 100 banks and the initial perturbation of 18 banks.

**Figure 19:**
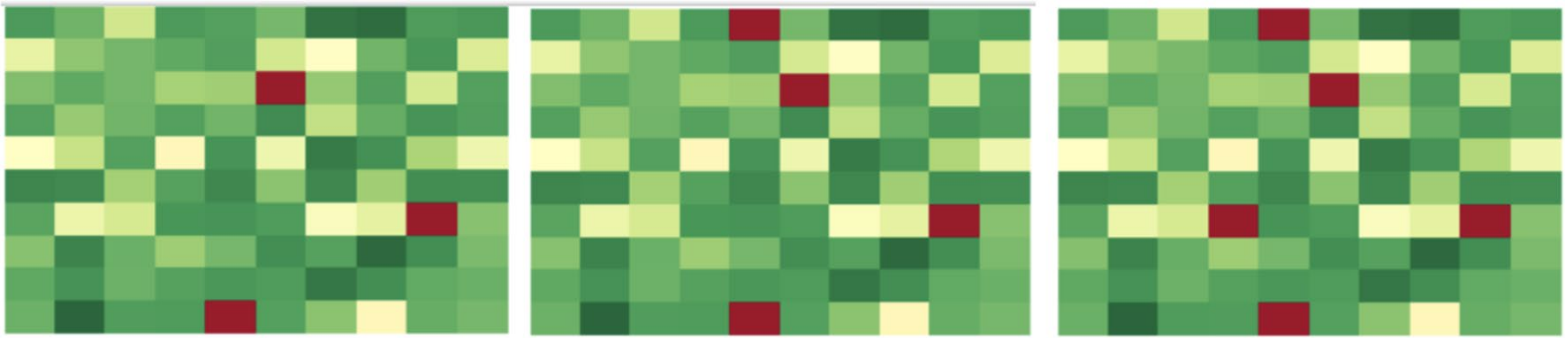
Heatmap visualization of cascade simulation for the two-stage optimized network.

The bank failures shown in red spread through the heatmap and contaminate the whole map in 8 iterations. In this case, 87 banks out of 100 fail, the cascade failure occurs, and the whole interbank network is failed. Figure 19 shows the cascade simulation with the same perturbation after we optimized the network using two-stage optimization with classical partitioning. The same perturbation no longer incurs cascade failure within the network and the simulation terminates after three iterations. As a result, only 5 out of 100 banks fail in the end and the cascade failure is completely mitigated for this interbank network.

## Experimental results with real-world data

To demonstrate the performance of our methodology with a real world network, we use the same data as in^13^ on cross-holdings of debt among nine countries (six Euro-based countries from^3^ plus Japan, the United Kingdom, and the United States). Table 1 shows the data for the total cross-holdings (in million $US ) extracted from the Q2 2015 report of the Bank of International Settlements^39^. The columns represent the country whose assets are being held and the rows represent the holding countries.

**Table 1:** Raw cross-holding matrix (non-normalized $C_0$).

	France	Germany	Greece	**Italy**	Japan	Portugal	Spain	UK	USA
France	0	158690	1330	285003	172268	13597	110840	234737	476762
Germany	186443	0	21040	97481	31165	15885	89294	415382	482631
Greece	1130	2047	0	347	52	33	101	11113	1797
Italy	43146	193754	805	0	5116	3232	43301	48056	32555
Japan	146676	89122	240	30469	0	539	20400	187862	1.31e06
Portugal	4435	2078	185	6242	242	0	12963	2758	2387
Spain	43548	89294	1001	46750	9510	63082	0	422592	257367
UK	172019	126033	4912	32537	98412	10512	28169	0	917815
USA	193151	152835	1429	60734	314551	5435	40910	477759	0

To convert the above matrix into the normalized cross-holdings matrix, $C _0$, we must first compute the self-holding matrix amount $\hat{ C }_0$ as mentioned in section "Interbank network value calculation". Using the assumption made by Reinhart and Kenneth^40^, we estimate the outside holding (debt) of a country to be 1/3 of the total holdings. Thus, the self-holdings are double the sum of outside holdings using the data from^39^. These values give $\hat{ C }_0$, which is then combined with the cross-holding matrix $C_0$ to form the original dependency matrix $A _0 = {\hat{C}}_0 ( I - C _0)^{-1}$ that appears in Table 2.

**Table 2:** Original dependency matrix ($A _0$).

	France	Germany	Greece	Italy	Japan	Portugal	Spain	UK	USA
France	0.68	0.04	0.00	0.05	0.04	0.00	0.02	0.06	0.11
Germany	0.04	0.68	0.00	0.02	0.02	0.00	0.02	0.09	0.11
Greece	0.03	0.04	0.67	0.01	0.01	0.00	0.01	0.17	0.07
Italy	0.04	0.12	0.00	0.67	0.01	0.00	0.03	0.06	0.06
Japan	0.03	0.02	0.00	0.01	0.69	0.00	0.01	0.05	0.19
Portugal	0.04	0.03	0.00	0.05	0.01	0.67	0.1	0.05	0.05
Spain	0.02	0.03	0.00	0.02	0.01	0.02	0.67	0.12	0.10
UK	0.04	0.03	0.00	0.01	0.03	0.00	0.01	0.70	0.17
USA	0.05	0.04	0.00	0.02	0.06	0.00	0.01	0.10	0.72

As an example on how to interpret the dependency matrix ($A _0$), we consider the following example: the entry in Table 2 from Portugal to USA represents the value of USA’s holdings of Portugal assets, and thus how much USA is harmed when Portugal debt loses value. The matrices of cross-holdings can be seen as weighted directed graphs, as shown in Figs. 21, 22, 23. The arrows show the way in which the losses flow from country to country during cascade simulations.

**Table 3:** Original primitive asset values *p* and the applied perturbation.

Country	Relative GDP (p)	Perturbation
France	12.29	3.082
Germany	16.81	0
Greece	1.02	1.02
Italy	9.30	1.267
Japan	20.00	0.396
Portugal	1.00	0
Spain	6.00	0.45
UK	12.99	0
USA	75.70	0

As in^3^, we assume that primitive assets *p* are proportional to the GDP (gross domestic product) of the countries and $D = I$. Normalizing Portugal’s 2015 GDP to 1 as shown in Table 3, the initial values in 2015 are $v_0=A_0 p$:

36 $$\begin{aligned} v_0&= \begin{bmatrix} 19.1755 \\ 22.5502 \\ 9.6336 \\ 14.3569 \\ 29.4733 \\ 7.3081 \\ 14.4945 \\ 23.8985 \\ 58.3153 \\ \end{bmatrix} \end{aligned}$$

To simulate the cascades, we applied the perturbation vector shown in the third column of Table 3. We randomly generated a perturbation vector with values ranging from 0 to the maximum relative primitive assets (relative GDP value) of each country. Then, we compute the debt value of each country at each iteration starting from the initial value of $v_0$. If the new value $v_i$ is less than 0.95 of its original value (critical value $v_c=0.95 v_0$), the country is said to have failed, and a discontinuous value penalty of 0.5 of the original bank’s value is applied ($b_i=v_i/2$).

**Figure 20:**
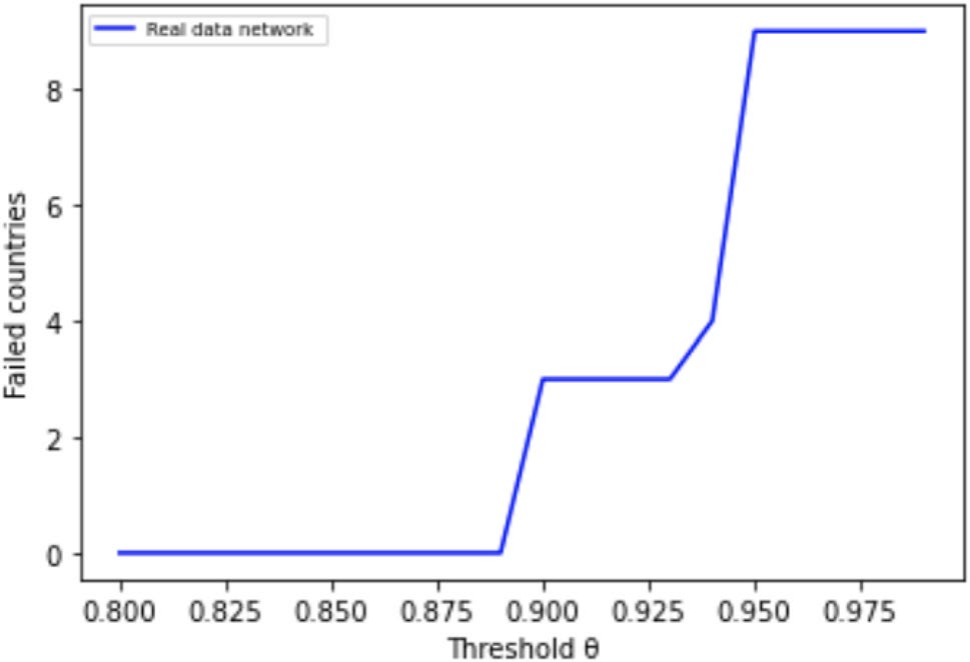
Simulation results showing failed countries relative to increasing values of critical threshold $\theta$. We use a high critical value threshold of $\theta = 0.95$ in our simulations to ensure that cascades are triggered and our algorithms are stress tested.

**Figure 21:**
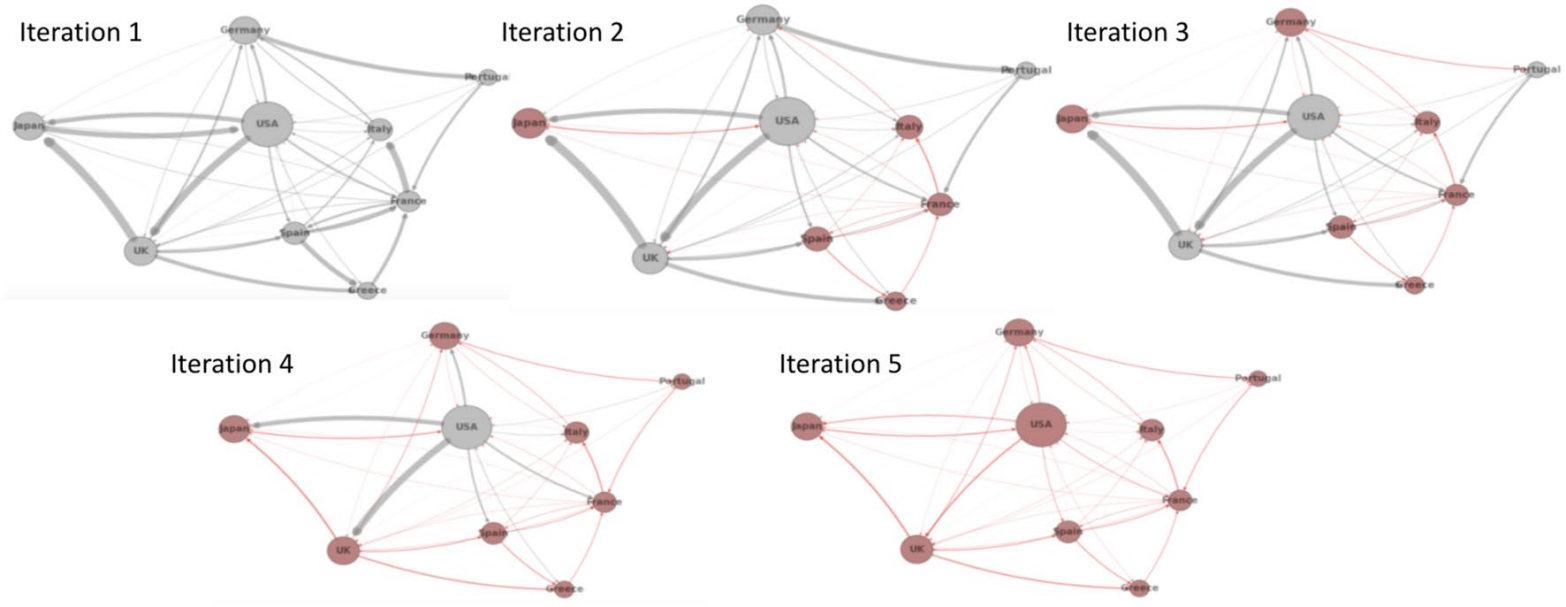
imulating cascades using real-world data.

**Figure 22:**
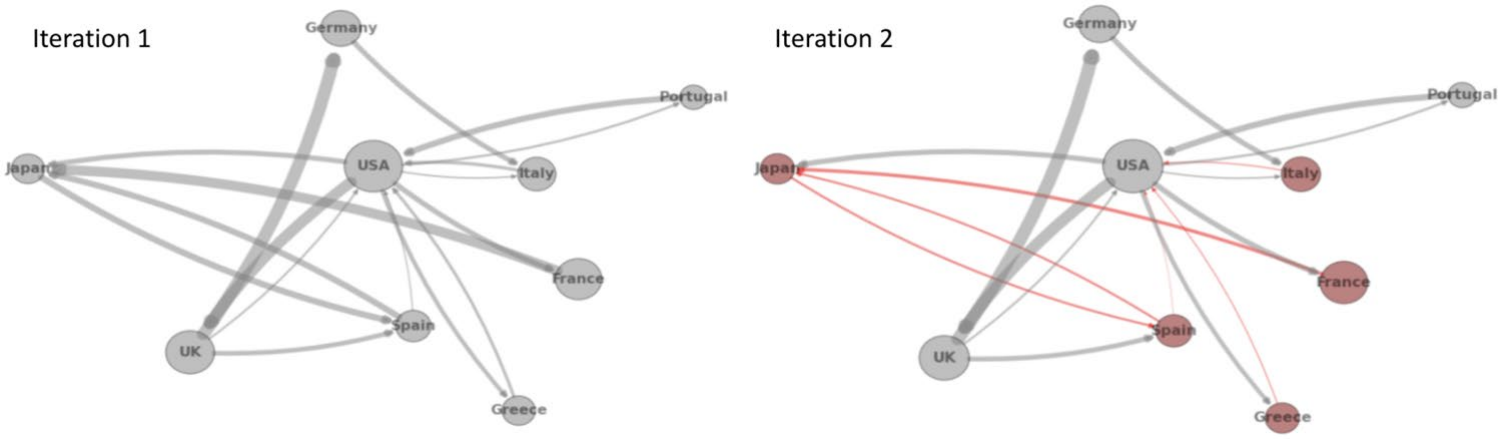
Simulating cascades using real-world data with one-stage optimization.

**Figure 23:**
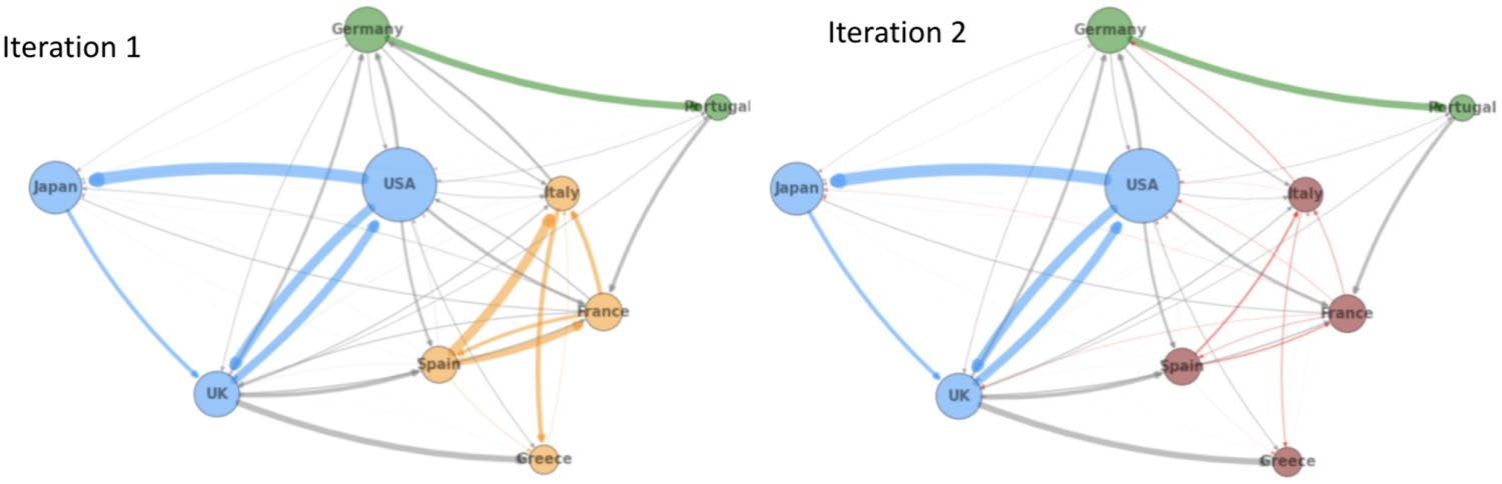
Simulating cascades using real-world data with two-stage optimization.

Figure 20 shows the results of running cascade simulations with multiple critical threshold values $\theta$ and computing the number of failures using the same real-world network data. Values of $\theta$ less than 0.88 result in no failures. We have intentionally chosen a high critical value threshold of $\theta = 0.95$ to ensure that cascades are triggered in our simulations and our algorithms are stress tested.

Figure 21 shows a simulation of cascades using real-world network data. After five iterations, all nine countries have failed. Figure 22 with one-stage optimization converges after one iteration with five failures. Figure 23 with two-stage optimization converges after one iteration with four failures that are localised within a cluster.

The performance with the real-world network data parallels the performance with synthetic data shown in section "Performance and scalability". The ability of two-stage optimization to confine contagion locally within the clusters will be beneficial as we scale up to more countries, therefore, we anticipate that the scalability demonstrated with the synthetic data will extend to real-world data.

## Conclusions

We experimented with a one-stage optimization algorithm based on a MILP formulation that globally optimizes the cross-holdings across the interbank network in order to minimize the total loss. Two problems with this algorithm were the scalability when the network grows and the changes to the connections (cross-holdings) were so drastic that the implementation in practice would not be realistic.

In order to address the scalability issue, we developed a two-stage optimization model using network partitioning with classical or quantum algorithms as the first stage. We developed new classical and quantum partitioning algorithms for directed and weighted graphs.

Using classical partitioning for our two-stage optimization, our experimental results show that our more computationally efficient two-stage algorithm is as good as one-stage in the sense of delaying cascade failures as we scale up to more nodes.

Using quantum partitioning for our two-stage optimization creates better partitions which further delays the phase transition of cascades and comes with additional computational efficiency benefits due to quantum superposition effects. However, we were only able to test up to 50 organizations due to quantum hardware limitations but quantum hardware will improve over time.

In addition, for both classical and quantum network partitioning algorithms, we found our two-stage optimization creates optimized networks with fewer and less drastic changes to cross-holdings that would be more realistic to implement. Furthermore, our experimental results show that minimizing the total possible loss is not equivalent to delaying cascade failures and demonstrate that optimizing cross-holdings locally within each module better confines the contagion and hence better delays cascade failures.

We used synthetic data and real-world data to assess our algorithms and show the benefits of our approach in mitigating systemic risk in financial systems combined with computational efficiency benefits. The real-world results aligned with our synthetic results and we demonstrated that our two-stage quantum algorithm is more resilient to financial shocks and delays the cascade failure phase transition under systemic risks with reduced time complexity.

## Data Availability

All data generated or analysed during this study are included in this published article and its supplementary information files.
